# Indigenous gut microbes modulate neural cell state and neurodegenerative disease susceptibility

**DOI:** 10.1016/j.cels.2025.101481

**Published:** 2026-02-03

**Authors:** Lisa Blackmer-Raynolds, Lyndsey D. Lipson, Anna Kozlov, Aimee Yang, Emily J. Hill, Maureen M. Sampson, Adam M. Hamilton, Isabel Fraccaroli, Sean D. Kelly, Pankaj Chopra, Jianjun Chang, Steven A. Sloan, Timothy R. Sampson

**Affiliations:** 1 Department of Cell Biology, Emory University School of Medicine, Atlanta, GA 30322, USA; 2 Department of Human Genetics, Emory University School of Medicine, Atlanta, GA 30322, USA; 3 Lead contact

## Abstract

The native microbiome influences numerous host processes, including neurological function. However, its impacts on diverse brain cell types remain poorly understood. Here, we performed single-nucleus RNA sequencing on the hippocampus of wild-type, germ-free mice, revealing the microbiome-dependent transcriptional landscape across all major neural cell types. We found conserved impacts on key adaptive immune and neurodegenerative transcriptional pathways. Mono-colonization with select indigenous microbes identified organism-specific effects on brain myeloid cell transcriptional state. *Escherichia coli* colonization induced a distinct myeloid cell activation state, increased brain-resident CD8^+^ T cells, and shaped amyloid phagocytic capacity, suggesting heightened disease susceptibility. Finally, *E. coli*-exposed 5xFAD mice displayed exacerbated cognitive decline and amyloid pathology, demonstrating the sufficiency of intestinal *E. coli* to worsen Alzheimer’s disease-relevant outcomes. Together, these results emphasize the broad, species-specific, microbiome-dependent consequences on neural cell states and highlight the capacity of specific microbes to modulate disease susceptibility.

## In brief

Blackmer-Raynolds et al. identify microbiome-dependent transcriptional signatures across all major neural cell types, including bacteria-specific responses within brain-resident myeloid cells. *Escherichia coli* colonization triggers adaptive neuroimmune activation and modulates neurodegenerative disease pathways in wild-type mice, translating to exacerbated disease outcomes in a mouse model of Alzheimer’s disease.

## Graphical Abstract

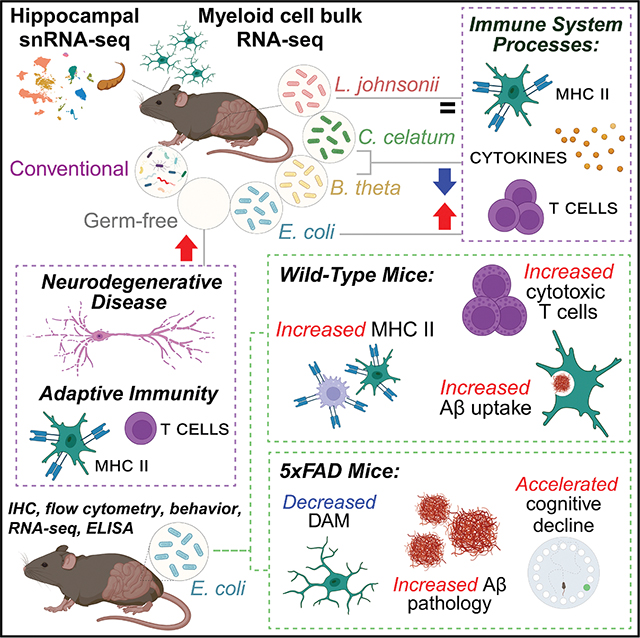

## INTRODUCTION

The human body is colonized by a diverse and complex community of microbes, the microbiome, that shapes a range of host physiological processes. An individual’s gastrointestinal (GI) tract harbors 100–500 unique bacterial species, with an estimated 3,500 unique strains of human gut-resident bacterial species worldwide, whose abundance and functions are influenced by genetic, environmental, and lifestyle factors. ^1–4^ Given this variability in microbiome composition between individuals, across the lifespan, and within the context of disease, understanding the physiological consequences of specific microbial taxa on health outcomes, particularly neurological disease, is essential.

Gnotobiotic mouse models raised in a sterile, germ-free (GF) environment, or treated with high-dose antibiotics, have provided insights into the contributions of the gut microbiome to host physiology. For instance, in the absence of indigenous microbes, GF mice have smaller, poorly developed lymphoid organs, limited lymphocyte maturation, and increased susceptibility to infectious diseases. ^5,6^ In the brain, GF or antibiotic-treated mice harbor immature microglia that are less capable of mounting inflammatory responses and defending against pathogens.^7–9^ In addition to—or perhaps even as a result of—microbiome contributions to neuroinflammatory tone, the microbiome also has broad impacts on neurological function in the context of both health and disease. A native microbiome is necessary for proper neurogenesis, myelination, and neurotransmitter production, with impacts on anxiety-like, social, and cognitive behaviors in mice.^10^ While some microbiome-dependent effects on immune and neurological functions are developmental and irreversible, others are quickly restored by microbial colonization or exposure to microbial metabolites, highlighting the importance of continuous microbial input for proper neurological function into adulthood.^5,8,11^

Whole microbiome manipulations in mouse models of disease have broad impacts on both behavioral and pathological outcomes.^10,12–14^ In the absence of an intact, native microbiome, mouse models of Alzheimer’s disease (AD) show significant improvements in cognitive performance and reductions in both amyloid beta (Aβ) and tau pathology.^15–22^ Depletion of myeloid cells from the brains of antibiotic-treated APPPS1–21 mice eliminates the protective effect of antibiotic treatment, suggesting that microbiome-mediated neuroimmune signaling contributes to pathological outcomes.^21^ While these studies emphasize the microbiome’s capacity to modulate overall brain health and function, how the microbiome shapes the transcriptional state across different brain-resident cell populations remains poorly understood. Furthermore, despite the various associations of specific bacterial taxa with neurological diseases,^12^ few studies have addressed the physiological contributions of individual microbes to neuroimmune functions and neurodegenerative disease susceptibility.

Here, we provide a single-cell characterization of the microbiome-dependent transcriptional state within the mouse brain and identify both the conserved and cell-type-specific transcriptional landscapes temporally modulated by microbiome-derived signals. In particular, the neuroimmune compartment was one of the most transcriptionally responsive. Evidence of microbiome-dependent immune signaling was found across cell types, suggesting that these cells act to propagate signals from the microbiome to other cells within the central nervous system (CNS). These responses are specific, as we find that select, non-pathogenic, gut-resident species are sufficient to uniquely shape the transcriptional state of brain-resident myeloid cells. In particular, the species *Escherichia coli* induces a distinct and temporally modulated transcriptional profile across not only brain-resident immune cells but also across neuronal and other glial populations. Colonization with *E. coli* dynamically regulates neurodegenerative disease-associated pathways across numerous neural cell types, including modifying myeloid cell phagocytic activity and T lymphocyte infiltration. Underscoring the pathological relevance of these disease-modifying, microbiome-dependent functions, oral exposure to *E. coli* worsens cognitive impairments and increases amyloid pathology within the 5xFAD mouse model of AD. Together, these results highlight the widespread impact of native microbiome-dependent signaling on the brain, as well as the specific consequences of individual gut microbes for neurological function, emphasizing the importance of the native microbiome in shaping transcriptional tone that can impact disease.

## RESULTS

### A complex microbiome is necessary for the steady-state transcriptional landscape of all major brain-resident cell types

**Figure 1.**
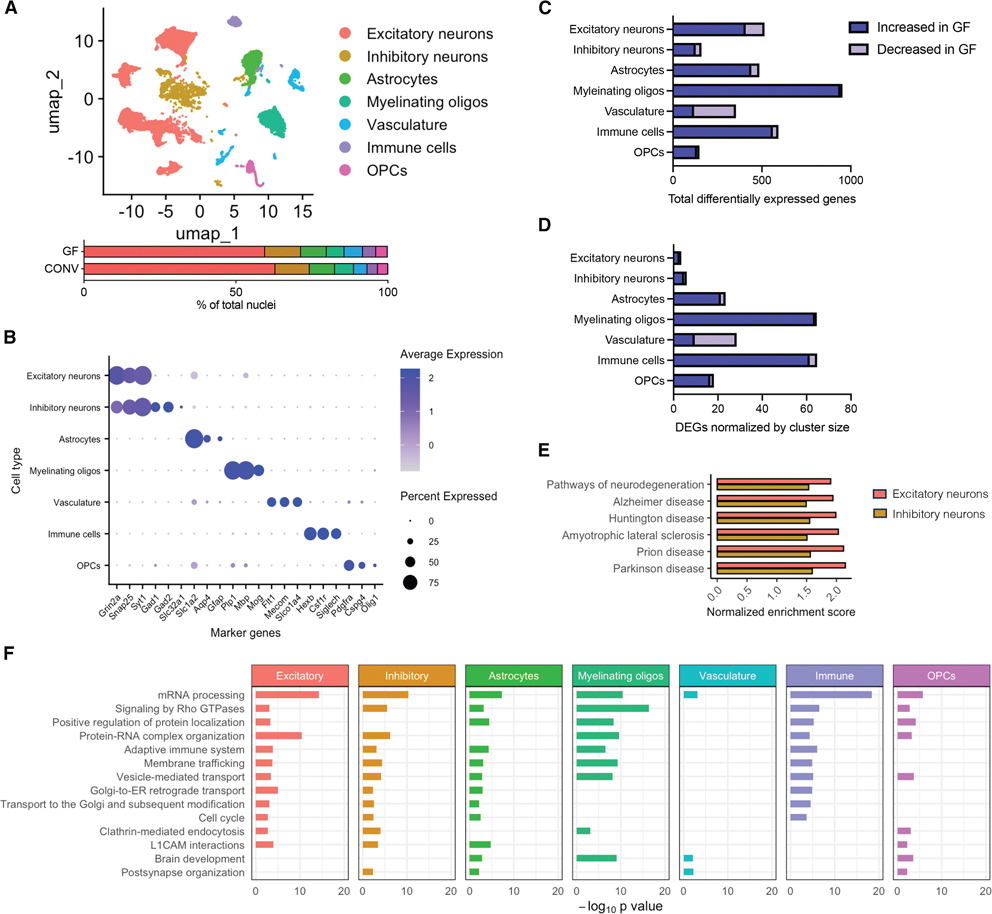
The microbiome shapes the transcriptional landscape of every major cell type in the brain Single-nucleus RNA-seq was performed on hippocampal samples from young adult female germ-free (GF) and conventionally raised (CONV) mice (Table S1; NIH GEO: GSE289589). (A) UMAP shows all major cell types identified as well as the amount of each cell type found within each treatment group. (B) Cell type markers representing each major cell-type cluster. (C) Total number of differentially expressed genes (DEGs; log fold change > |1|, *p* < 0.001) in GF mice compared with CONV across each cell type. (D) DEGs normalized by the number of cells in that cluster $\left(\frac{\text{DEGs}}{\text{total cells in cluster}}\times 100\right)$. (E) GSEA showing increased neurogenerative disease KEGG pathways in GF excitatory and inhibitory neurons. (F) Representative pathways increased in at least four cell types by overrepresentation analysis. Data represent cells from 4 mice per treatment.

In order to understand how select gut bacterial species impact the brain, we first sought to determine how the complete absence of an indigenous microbiome influences the cellular transcriptional landscape. We performed single-nucleus RNA sequencing (snRNA-seq) on hippocampal tissues derived from young adult female GF or conventional (CONV) mice born and raised with a complete and intact microbiome. Unsupervised clustering of 23,963 total nuclei (representing four mice per treatment group) revealed seven unique clusters, roughly equally represented across each microbiome status (Figure 1A), that were identified as the following cell types based on marker genes^23^: excitatory neurons (cluster 1), inhibitory neurons (cluster 2), astrocytes (cluster 3), myelinating oligodendrocytes (cluster 4), vasculature (cluster 5), immune cells (cluster 6), and oligodendrocyte progenitor cells (OPCs; cluster 7) (Figures 1B and S1A; Table S1). Differential gene expression analysis demonstrated microbiome-dependent transcriptional responses within each cluster, with most differentially expressed genes (DEGs) occurring within myelinating oligodendrocytes, immune cells, excitatory neurons, and astrocytes (Figure 1C; Table S1). Upon normalization by cluster size, to account for the increased power in highly abundant clusters, myelinating oligodendrocytes and immune cells were found to have the largest microbiome-dependent transcriptional response (Figure 1D). This highlights both the broad effects of microbiome-derived signaling and the unique susceptibility of specific cell types that may represent central modulators of microbiome-mediated signaling to the brain.

To evaluate the biological relevance of the identified microbiome-dependent DEGs, we performed pathway analysis across cell types (Figures 1E, 1F, and S1B; Table S1). We first examined biological pathways conserved in their microbiome-dependent responses across at least four cell types. While we did not observe any pathways decreased within four cell types, there were a number of shared microbiome-dependent biological pathways enriched across multiple cell types (Figure 1F). Among all cell clusters, mRNA processing (Gene ontology [GO]:0006397) was highly enriched in the absence of a microbiome. Across four to five cell types, we observed a further enrichment in protein localization (GO:0010954) and membrane trafficking (R-MMU-199991) pathways, adaptive immune system (R-MMU-1280218), cell cycle (R-MMU-1640170), and brain development (GO:0007420) pathways, indicating a widespread repression of these particular signaling pathways by the native microbiome. These observations align with prior, targeted studies that demonstrated microbiome-dependent impacts on brain development and organization^10^ and neuroimmune activation.^7–9^ Even within the pathways that were increased in fewer than four cell types, impacted pathways were conceptually related, falling broadly within the categories of development, cellular stress, and immune processes (Figure S1B). Within both excitatory and inhibitory neuron clusters, gene set enrichment analysis (GSEA) of KEGG pathways revealed a microbiome-dependent enrichment of major neurodegeneration-associated KEGG pathways (Figure 1E). These same cell clusters also displayed increased immune-related pathways (e.g., adaptive immune system [R-MMU-1280218], neutrophil degranulation [R-MMU-6798695], cellular response to interleukin-4 [GO:0070670], and interferon signaling [R-MMU-913531], among others), emphasizing the ability of the indigenous microbiome to shape the neuronal transcriptional landscape and modulate neuroimmune and neurodegenerative disease pathways (Figure S1B).

### Select gut microbes differentially and specifically modulate the brain-resident myeloid cell transcriptome

**Figure 2.**
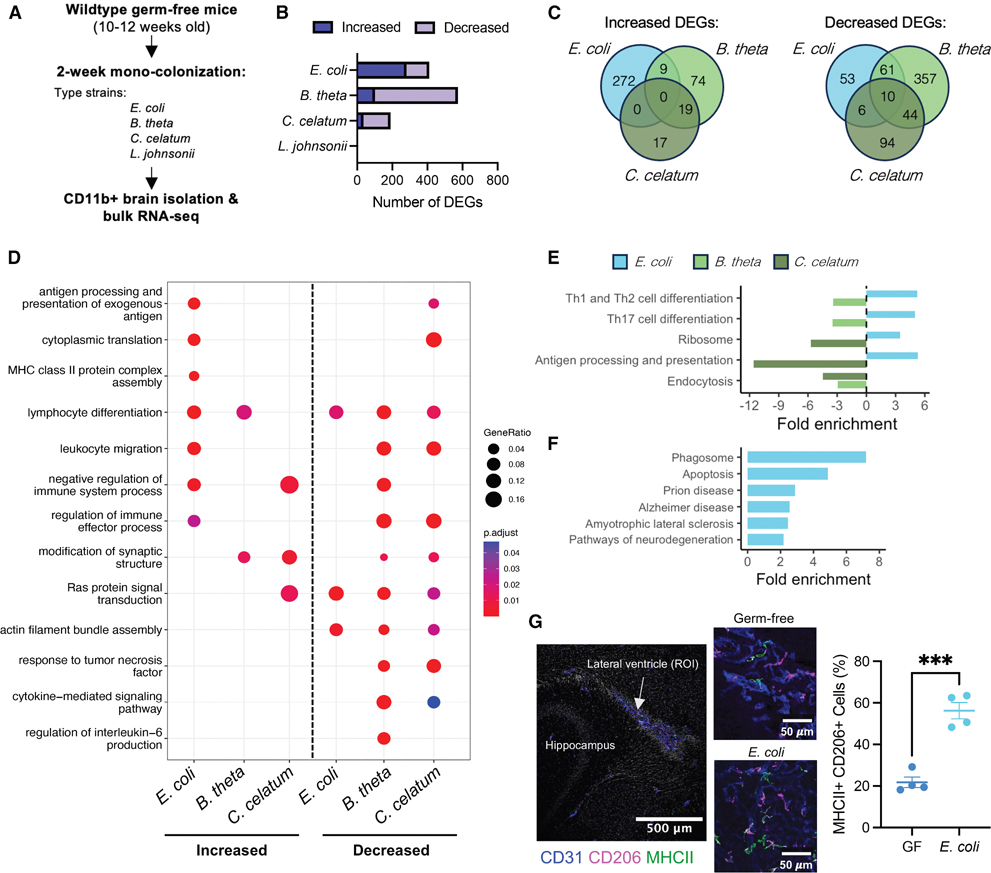
Select gut bacteria uniquely shape neuroinflammatory tone (A) Young adult wild-type germ-free (GF) mice were mono-colonized with type strains of interest for 2 weeks: *Escherichia coli* (*E. coli*), *Bacteroides thetaiotaomicron* (*B. theta*), *Clostridium celatum* (*C. celatum*), and *Lactobacillus johnsonii* (*L. johnsonii)*. After mono-colonization, CD11b^+^ myeloid cells were isolated from the brains, and bulk RNA-seq was performed (Table S2; NIH GEO: GSE289591). (B) Number of differentially expressed genes (DEGs; log fold change > |0.25|, *p* < 0.05) in mono-colonized mice compared with GF. (C) Venn diagrams showing overlapping DEGs across colonization states. (D) Comparison of overrepresentation-based pathway analysis results across mono-colonization states. (E) KEGG pathway analysis was run using DEGs for each treatment. Pathways that were significantly increased or decreased in at least two treatments are shown. (F) KEGG pathways increased specifically after *E. coli* mono-colonization. (G) Immunohistochemistry was performed on the choroid plexus of the lateral ventricle to quantify the percentage of CD206^+^ cells that were also MHC class II^+^. Representative images highlight the region of interest (ROI) analyzed (left) and example zoomed-in images used for quantification for each treatment (right). Quantification was performed using at least two images per mouse and four mice per treatment group. *n* = 3–7 mice. In (G), dots represent individual mice, and error bars represent mean ± SEM. *** *p* < 0.001.

Brain-resident immune cells are particularly susceptible to perturbations within the gut microbiome^7–9^ (Figures 1C and 1D). Further, consistent increases in immune-related pathways across non-immune brain cell types suggest that immune cells are critical for transducing microbiome-derived signaling to other cells within the brain. However, it is unclear whether these activities are a generalized response to broad bacterial organisms or if specific, indigenous microbes are sufficient to impart differential effects on brain-resident immune cells. Therefore, we set out to test the specificity and sufficiency of select, indigenous gut bacterial species to modulate the transcriptional state of brain-resident myeloid cells. Young adult, wild-type GF mice were mono-colonized with select bacterial type strains representing prevalent genera within the mammalian gut microbiome—*Bacteroides thetaiotaomicron*, *Clostridium celatum*, *Lactobacillus johnsonii*, and *Escherichia coli*—for 2 weeks. Immunopanning was performed to enrich for CD11b^+^ myeloid cells (largely representing microglia but also present on other leukocytes^24^), followed by bulk transcriptomics comparing GF mice to each mono-colonized condition (Figure 2A).

Validating myeloid cell enrichment, the most highly expressed genes within the dataset included canonical microglia genes such as *Hexb*, *Csf1r*, *P2ry12*, and *Tmem119* (Table S2). Differential gene expression analysis demonstrated that all but one of the bacteria studied—*L. johnsonii*—were sufficient to modulate myeloid cell gene expression, with colonization by *B. thetaiotaomicron* and *E. coli* inducing the most DEGs (Figure 2B; Table S2). Comparison of the DEGs increased and decreased after mono-colonization shows very few overlapping DEGs, highlighting the specificity of the transcriptional response to each of these unique bacterial species (Figure 2C). No shared DEGs were increased by all three species, and of the decreased genes, only ten DEGs (less than 2%) were shared across colonization states (Figure 2C). In addition, pathway analysis comparing across each bacterial colonization state shows near inverse effects between the unique species (Figure 2D; Table S2). Where *E. coli* induced an increase in pathways involved in immune activation and adaptive immune responses (in a mixed-sex cohort), *B. thetaiotaomicron* and *C. celatum* decreased many of these same pathways in male mice (with no DEGs detected in female mice). Further emphasizing the unique transcriptional response of brain myeloid cells to *E. coli* compared with the other bacterial species tested, KEGG pathway enrichment revealed that *E. coli* mono-colonization triggered an inverse effect to that of *B. thetaiotaomicron* or *C. celatum* amongst all shared KEGG pathways (Figure 2E). In addition, *E. coli* alone induced an increase in KEGG pathways involved in neurodegenerative diseases—including prion disease (mmu05020), AD (mmu05010), and amyotrophic lateral sclerosis (ALS, map05014)—highlighting the potential for *E. coli* colonization to induce a disease-susceptible transcriptional state within the brain (Figure 2F).

Lipopolysaccharide (LPS)—a component of the outer membrane of Gram-negative bacteria—is known to stimulate neuroimmune activation; however, GSEA of genes within the “response to LPS” pathway (GO:0032496) showed no significant increase in this gene set in any mono-colonization state (Figures S2A and S2B), suggesting that the response observed within CD11b^+^ cells is not solely due to microbial LPS stimulation. In fact, *B. thetaiotaomicron* (which produces LPS) and *C. celatum* (which does not) both show a significant negative normalized enrichment score (Figure S2B), indicating that this pathway is repressed after mono-colonization. In line with this, multiplex measurement of cytokines in both intestinal tissues and serum demonstrated limited impacts of any of the bacteria studied on inflammatory cytokine or chemokine levels (Figure S2C). While a decrease in ileal tumor necrosis factor (TNF) and an increase in colonic CXCL1 and TNF were observed in most mono-colonized conditions (Figure S2C), the only bacteria-specific response was reduced IL-1β following *L. johnsonii* colonization, which did not associate with an altered brain-resident CD11b^+^ cell state. In addition, we observed no overt effects on colon length, expression of intestinal tight junction protein genes *Cldn5*, *Ocln*, and *Tjp1* (ZO-1), or other measures of GI function between GF and mono-colonized mice (Figures S2D–2I), suggesting that intestinal inflammation was limited and not unique to any one bacterial species tested. Importantly, there was no observed effect on circulating cytokines/chemokines at this time point of colonization by any tested microbe, suggesting no overt systemic inflammation (Figure S2C). In total, these data indicate that the observed unique transcriptional impacts within the brain and CD11b^+^ cells are likely not due to overt inflammatory responses upon colonization.

Recent studies^25–29^ have emphasized the sufficiency of the microbiome to modulate adaptive immune processes within the brain. Given our data demonstrating unique neuroimmune states induced by select microbes, we therefore sought to determine whether the observed increase in adaptive immune processes within brain myeloid cells after *E. coli* mono-colonization translated to functional outcomes. Specifically, we sought to validate the transcriptional increase in genes involved in major histocompatibility complex class II (MHC class II) antigen presentation by performing immunohistochemistry for MHC class II within the brain of male mono-colonized mice (Figure 2G). MHC class II signal was not robustly detected in the brain parenchyma but instead was primarily found within CD206^+^ cells, canonically considered to be either border-associated macrophages (BAMs) or infiltrating myeloid cells, within the CD31^+^ choroid plexus of the lateral ventricle. In line with the transcriptional data, when compared with GF mice, *E. coli* mono-colonization triggered more MHC class II^+^/CD206^+^ double-positive cells (Figure 2G), demonstrating that the observed increase in adaptive immune transcriptional processes translates to altered MHC class II production within specific subpopulations of myeloid cells at the interface between the central and peripheral immune systems. However, no change in IBA1^+^ area (within the dorsal hippocampus, important for learning and memory^30^) was observed, even within *E. coli* mono-colonized mice (Figure S2J), demonstrating that *E. coli* colonization induces more subtle impacts on neuroimmune activation state, not overt gliosis.

### *E. coli* elicits a temporal transcriptional response across CNS-resident cells

**Figure 3.**
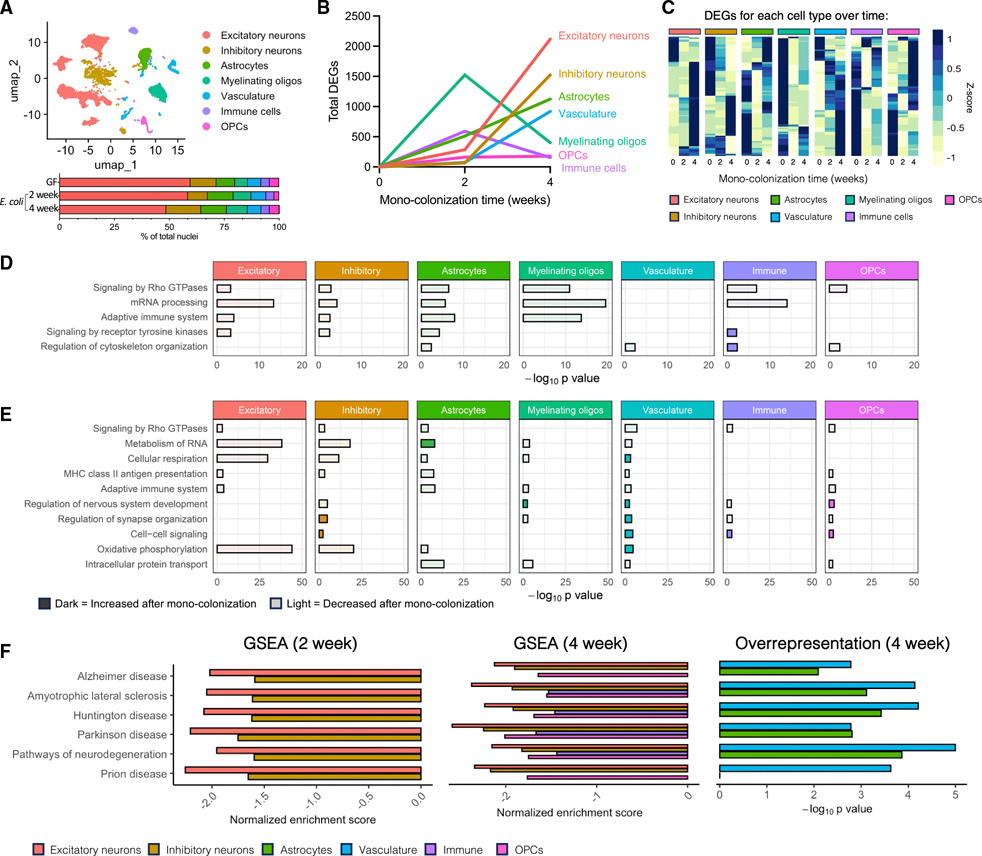
Gut colonization with E. coli temporally modulates gene expression across all cell types the brain snRNA-seq was performed on hippocampal samples from germ-free (GF) mice and mice that were mono-colonized with *E. coli* for either 2 or 4 weeks (Table S3; NIH GEO: GSE289589). (A) UMAP showing cell clusters and a bar graph showing the percentage of total nuclei within each cluster based on treatment. (B) Graph showing the total number of differentially expressed genes (DEGs; log fold change > |1|, *p* < 0.001) compared with GF per cell type across time. (C) Heatmap shows relative expression levels (as a *Z* score) of all DEGs (at either 2 or 4 weeks) within each cell type across time (note each heatmap represents only that cell type’s DEGs). Overrepresentation pathway analysis was run using the DEGs between GF and *E. coli* mono-colonized mice at each time point. (D and E) Increased and decreased pathways that overlapped in at least four cell types at 2 weeks are shown in (D) and at 4 weeks are shown in (E). Gene set enrichment analysis (GSEA) was also performed to identify neurodegeneration KEGG pathways that are modulated by *E. coli* colonization. (F) Shows the normalized enrichment score for each cell type with a significant GSEA result at 2 and 4 weeks as well as neurodegeneration KEGG pathways increased in overrepresentation analysis at 4 weeks. Data are from 4 mice per treatment.

*E. coli* colonization has demonstrated contributions to pathologies in models of neurodegenerative disease,^31–34^ and increased abundances of *Enterobacteriaceae* (the bacterial family that includes *E. coli*) are reported in persons with neurodegenerative disorders.^12,35–39^ Given our observations here that this microbe is sufficient to drive both adaptive immune and neurodegenerative disease-associated pathways in brain-resident myeloid cells, we next sought to understand how this organism dynamically shapes transcriptional tone across neural cell types. We performed snRNA-seq on hippocampal samples from young adult female mice mono-colonized with *E. coli* for either 2 or 4 weeks to capture both short- and longer-term transcriptional responses following microbial colonization in comparison to GF controls. Clustering of 30,093 total nuclei (representing four mice per treatment group) was performed in combination with previously assessed nuclei (Figure 1A) to create comparable cell clusters with no statistically significant differences in cell abundances between treatments (Figures 3A and S3A; Table S3).

Differential gene expression analysis demonstrated robust time-dependent shifts in the number of DEGs in each cell type compared with GF (Figures 3B, 3C, and S3B; Table S3). Similar to the microbiome-dependent effects we previously observed (Figure 1), we found that immune cells and myelinating oligodendrocytes displayed the largest initial transcriptional response to colonization (Figure 3B). However, the number of DEGs in both of these cell types was dramatically reduced at 4 weeks of colonization, and many of those initial DEGs appeared to return to a pre-colonized state (Figure 3C). This, however, was not a complete restoration, suggesting that colonization can lead to subtle yet longer-lasting transcriptional states that may influence future cellular outcomes. In contrast to immune cells and myelinating oligodendrocytes, the number of DEGs in the other cell clusters (excluding OPCs that remained comparatively unresponsive) was dramatically higher at 4 weeks compared with 2 weeks. For example, the neuron clusters—with the highest number of DEGs at 4 weeks—displayed a limited transcriptional response until 4 weeks post-colonization with *E. coli* (Figures 3B and 3C). We interpret this to suggest that these cells are either slower to respond to microbiome status or, instead, respond to those signals derived from more acutely responsive cells.

To better understand the breadth of the transcriptional response to *E. coli* colonization, we performed pathway analysis within every cell cluster at each time point, as well as within four additional sub-clusters of neuronal cell types (Figures 3D, 3E, S3C, S3D, and S3E; Tables S3 and S4). Neuronal sub-clustering based on cell marker genes^23^ identified four distinct clusters representing CA1/CA3 excitatory neurons (cluster 1), dentate gyrus excitatory neurons (cluster 2), inhibitory neurons (cluster 3), and NP-CT-L6b excitatory neurons (Figure S3C; Table S4). While differential gene expression and pathway analysis revealed some cell-type-specific responses (Table S4), as a whole, similar patterns were observed across the broader neuron populations, so excitatory neuron sub-clusters were grouped together during subsequent analyses.

To evaluate conserved biological processes shared across cell types, pathways that were significantly altered across at least four distinct cell types at each time point were quantified. At both 2 and 4 weeks post-colonization, we observed a decrease in pathways involved in Rho GTPase signaling, RNA metabolism, and adaptive immunity across cell types (Figures 3D and 3E). This mirrors our observations in mice with a complex/intact microbiome (Figure 1F) and highlights that these transcriptional pathways may be highly sensitive to microbial colonization. By contrast, pathways involved in nervous system/brain development were cell-specifically modulated in *E. coli*-colonized mice, while these were consistently decreased in CONV mice compared with GF animals (Figure 1F), suggesting that *E. coli* is not simply inducing a more CONV-like state within the brain. Cell-specific pathways further highlight that colonization with *E. coli* induced a time-dependent response in immune, cellular stress, and cellular organization/transport pathways (Figures S3D and S3E).

Because these transcriptional responses are relevant for neurological disease, we specifically examined neurodegenerative disease KEGG pathways by both GSEA and overrepresentation pathway analysis (Figure 3F). We observed a modulation of neurodegeneration KEGG pathways across nearly every cell type following *E. coli* colonization, with a decrease in KEGG pathways of neurodegenerative diseases in both excitatory and inhibitory neurons following colonization across both timepoints, and in microglia and OPCs by 4 weeks post-colonization (Figure 3F). In addition, overrepresentation-based pathway analysis demonstrated an enrichment of neurodegeneration pathways in both astrocytes and vasculature at 4 weeks of colonization (Figure 3F). Taken together, these results emphasize a link between *E. coli* colonization and transcriptional pathways that are involved in the modulation of neurodegenerative disease risk.

### *E. coli* dynamically shapes neuroimmune state, including phagocytic capacity and T cell infiltration

**Figure 4.**
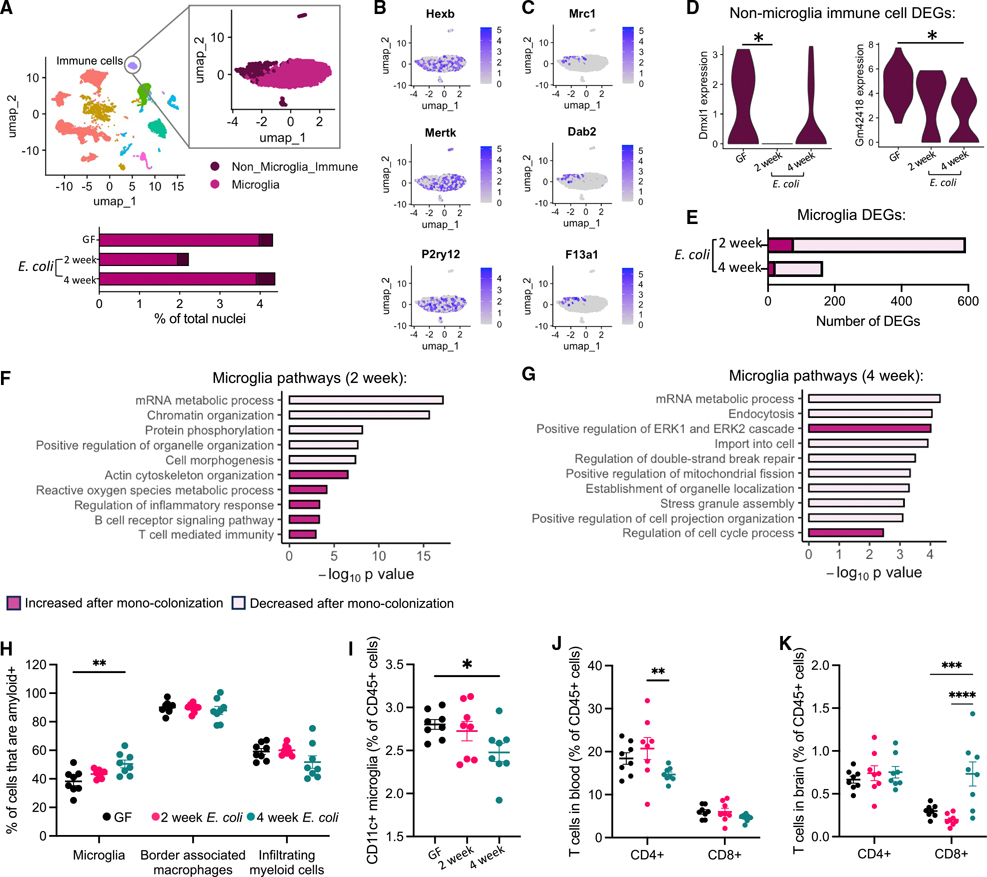
E. coli colonization temporally regulates neuroimmune activation The immune cell cluster identified in Figure 2 was further sub-clustered to distinguish microglia from non-microglia immune cells (Table S5; NIH GEO: GSE289589). (A) Shows cell clustering UMAP and % of all nuclei within each cell type by treatment group. (B and C) Markers defining each cell cluster are overlaid upon the immune UMAP, with (B) representing microglia markers and (C) representing non-microglia markers. Differential gene expression analysis was performed on both cell clusters. (D) Shows violin plots of the two differentially expressed genes (DEGs) that reached significance (*p* < 0.05) in the low-powered non-microglia immune cluster. (E) Shows the number of DEGs that reached significance (log fold change > |1|, *p* < 0.001) in the microglia cluster at each time point. (F and G) (F) Pathway analysis of microglia DEGs after 2 weeks of *E. coli* mono-colonization and (G) after 4 weeks. (H) Flow cytometry was used to evaluate the uptake of fluorescent amyloid by microglia (CD45^+^, CD11b^+^, Ly6C^−^, and CD38^−^), BAM (CD45^+^, CD11b^+^, LY6C^−^, and CD38^+^), and infiltrating myeloid cell (CD45^+^, CD11b^+^, and Ly6C^+^) populations. (I) Quantification of CD11c+ microglia, a marker of DAMs. (J and K) Quantification of CD4^+^ and CD8^+^ T cells (CD45^+^ and CD3^+^) within (J) the blood and (K) the brain. For (A–G), cells represent 4 mice per treatment. For (H–I) *n* = 8, dots represent individual mice, and error bars represent mean ± SEM. * *p* < 0.05, ** *p* < 0.01, *** *p* < 0.001, **** *p* < 0.0001.

Since we observed the immune cell cluster as having the most increased DEGs at 2 weeks post-*E. coli* colonization (Table S3), and bulk RNA sequencing (RNA-seq) of brain myeloid cells displayed a microbiome-dependent adaptive immune and neurodegenerative disease phenotype (Figure 2), we sought to further characterize microglia responses as potential mediators of microbiome-derived signaling in the brain. In order to distinguish the microbiome-dependent effects on microglia compared with other immune cell types, we performed sub-clustering and pathway analysis within the immune cell cluster (Figures 4A–4G; Table S5). Unsupervised sub-clustering identified two main sub-clusters: microglia and non-microglia immune cells (e.g., BAMs and infiltrating peripheral immune cells) (Figures 4A–4C). While similarly prevalent irrespective of colonization status, non-microglia immune cells were lowly abundant and showed little transcriptional responsiveness to *E. coli* across both time points (Figure 4D); however, this may be due to an insufficient sample size, rather than a lack of susceptibility to perturbation.

By contrast, differential gene expression analysis identified 515 repressed and 78 induced genes in microglia following 2 weeks of mono-colonization (Figure 4E). Pathway analysis on these DEGs identified similar pathways as we observed in our analysis of CD11b^+^ cells (Figure 2), including an increase in pathways involved in inflammatory responses and adaptive immunity, whereas downregulated pathways included those characterized as cellular organization and mRNA processing (Figure 4F). At 4 weeks post-colonization, differential gene expression analysis of microglia revealed 23 increased and 142 decreased DEGs (Figure 4E). While we observed a consistent repression in genes involved in mRNA metabolic processes and cellular organization, as at the 2-week time point, microglia also showed a decrease in cellular stress pathways (e.g., regulation of double-stranded break repair [GO:2000779] and stress granule assembly [GO:0034063]) at 4 weeks post-colonization (Figure 4G).

We next sought to determine whether *E. coli*-dependent transcriptional responses in immune cells resulted in relevant, functional outcomes within these cell types. We first evaluated phagocytic activity in response to a disease-relevant stimulus, fibrillized human Aβ 1–42, in an *ex vivo* assay. Single-cell suspensions of brain tissue were exposed to fluorescently labeled Aβ 1–42 fibrils, and uptake across brain myeloid cell populations was measured by flow cytometry (Figures 4H and S4A). While we did not find any difference in overall abundance of microglia in the brain following *E. coli* colonization (Figure S4B), we did observe a substantial impact to their phagocytic capacity (Figure 4H). At 2 weeks post-colonization, amyloid uptake remained at control levels across myeloid cell populations. However, at 4 weeks post-colonization, microglia displayed increased phagocytic capacity (Figure 4H). This effect was specific to microglia, as we observed no impact to amyloid uptake by BAMs or infiltrating myeloid cells, despite a decrease in overall BAM levels within the brain at both timepoints (Figures 4H and S4B). Interestingly, we also observed a time-dependent decrease in CD11c^+^ microglia, a marker of disease-associated microglia (DAMs)^40^ (Figure 4I), further implicating the ability of *E. coli* to modulate disease-relevant processes in these cells. Together, these results emphasize that *E. coli* colonization induces functional impacts on microglia, which alters their responsiveness to disease-relevant stimuli.

Our data show that gut colonization by *E. coli* induces adaptive immune pathways across neural cell types (Figures 2 and 3), in conjunction with an increase in MHC class II protein levels (Figure 2G) and increased phagocytosis (Figure 4H). We therefore hypothesized that *E. coli* colonization would trigger activation and infiltration/proliferation of adaptive immune cells within the brain, namely T lymphocytes. To assess this possibility, we measured CD4^+^ and CD8^+^ T cells by flow cytometry in both the brain and in circulation. In the blood, we observed a decrease in CD4^+^ T cells at 4 weeks post-colonization, with no observable effects on CD8^+^ T cells or circulating myeloid cells (Figures 4J and S4C). By contrast, within the brains of mice mono-colonized with *E. coli* for 4 weeks, we found a significant increase in the abundance of CD8^+^ T cells, with no observable effect on CD4^+^ cell abundances (Figure 4K). These data demonstrate that the transcriptional responses induced by *E. coli* colonization result in functional neuroimmune impacts that shape phagocytic capacity and CD8^+^ T cell infiltration into the CNS.

### *E. coli* modulates cognitive impairment in an animal model of amyloid pathology

**Figure 5.**
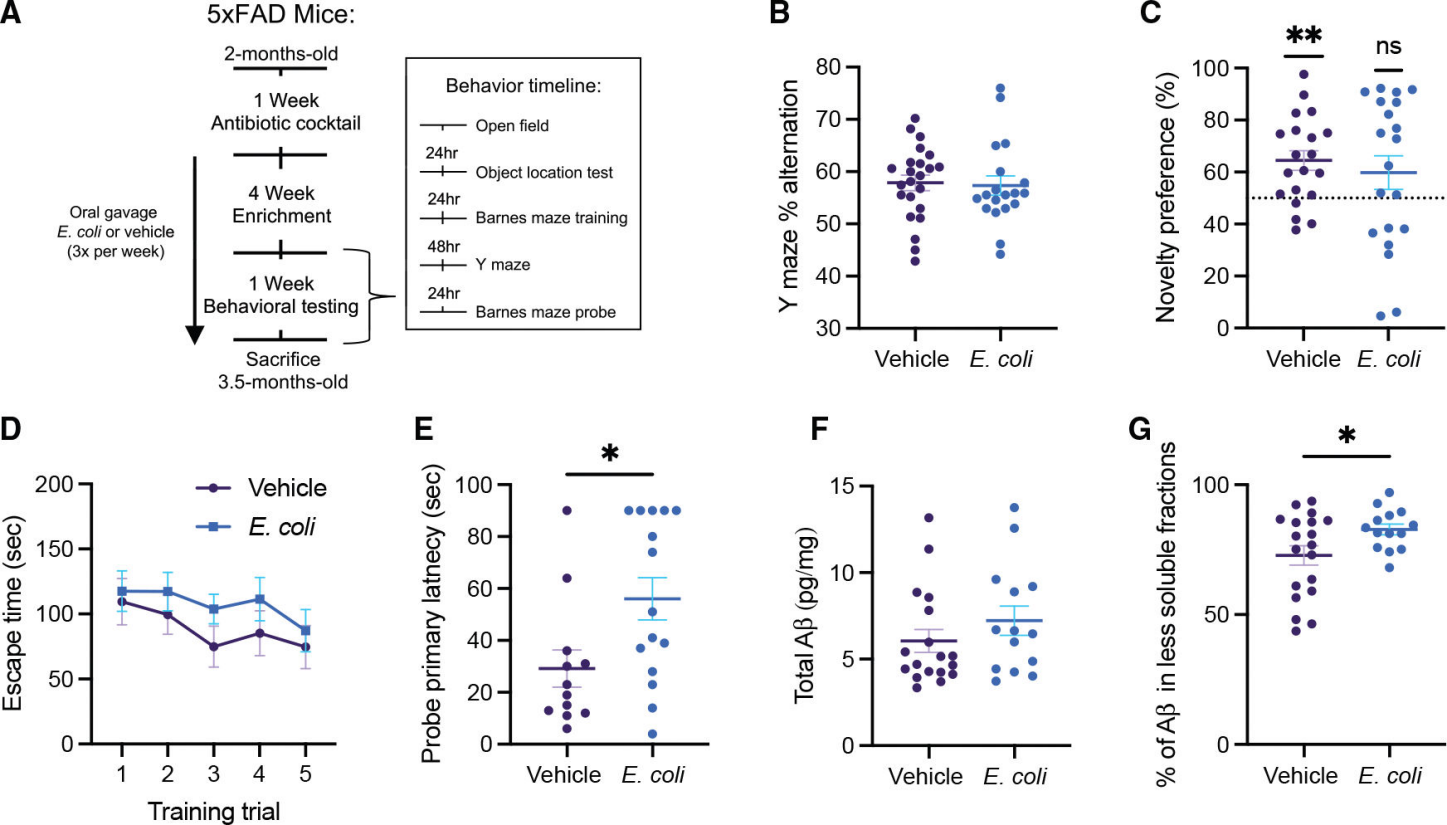
*E. coli* exposure exacerbates AD outcomes in 5xFAD mice (A) Male and female 2-month-old 5×FAD mice underwent a 1-month enrichment paradigm followed by behavioral and pathological assessments. (B–E) Behavioral testing was performed on the (B) Y maze, (C) object location test, (D) Barnes maze training, and (E) Barnes maze 72-h probe trial to evaluate different forms of spatial memory function. (F and G) Levels of Aβ in the hippocampus were measured within Tris, Triton, and formic acid soluble protein fractions, with total Aβ reported in (F) and the percentage of Aβ found within the less soluble Triton and formic acid fractions reported in (G). *n* = 11–15; dots represent individual mice, and bars represent mean ± SEM. Groups were compared using a two-tailed *t* test except in (C), where each condition was also compared with the 50% chance level using a one-sample *t* test. * *p* < 0.05, ** *p* < 0.01; ns = not significant based on one-sample *t* test. For the object location test, the *R*^2^ value = 0.012; for the Barnes maze probe primary latency, the *R*^2^ value = 0.189.

We have demonstrated that *E. coli* is sufficient to transcriptionally modulate adaptive immune and neurodegenerative disease pathways across many cell types in the brain. Notably, *E. coli* and closely related organisms in the *Enterobacteriaceae* family are reported to be enriched within the gut microbiome of individuals with neurodegenerative disease,^12,35–39^ suggesting they may contribute to disease processes. In addition, increased CD8^+^ T cells have been reported within the brains of individuals with AD and have been shown to exacerbate AD-like pathology in mouse models.^41–45^ We therefore sought to directly test the disease-modulatory potential of *E. coli* within the context of AD, the most prevalent neurodegenerative disease. To evaluate the sufficiency of GI *E. coli* to modulate AD outcomes, we used the well-characterized 5xFAD mouse model,^46^ which displays amyloid pathology beginning at 2 months of age^47^ and cognitive impairment between 3 and 6 months of age.^48^ 2-month-old CONV 5xFAD mice were orally exposed to ∼10^8^ colony-forming units (CFUs) of non-pathogenic *E. coli* (as in our mono-colonization experiments) or vehicle control three times a week for 4 weeks prior to behavioral and pathological assessments (Figure 5A). This timing allowed for a test of exacerbation over this model’s early pathological progression. Irrespective of treatment, 5xFAD mice displayed similar outcomes in both the open field and intestinal behaviors, suggesting that *E. coli* exposure did not induce overt sickness or anxiety-like behaviors, and GI functions were not robustly disrupted (Figures S5A–S5E). While working memory—as measured in the Y maze test—appeared intact (Figure 5B), *E. coli* exposure resulted in a loss of novelty preference in the object location test (Figure 5C). While the vehicle control mice displayed a significant novelty preference (compared with the 50% chance level, *R*^2^ = 0.43), the difference between vehicle control and *E. coli*-treated 5×FAD mice did not reach significance, likely due to the significantly higher variability in the *E. coli-*treated animals (*F* = 2.9). While learning capacity during Barnes Maze training was not impacted (Figure 5D), *E. coli-*treated 5×FAD mice displayed a longer primary latency during the probe trial, suggesting a loss of typical memory function (Figure 5E; *R*^2^ = 0.19). These observations were specific to the 5×FAD genotype, as wild-type littermates did not demonstrate any loss of cognitive functions in an identical battery of tests (Figures S5F–S5J). Thus, in a genotype-specific fashion, exposure to intestinal *E. coli* is sufficient to accelerate the development of cognitive decline in this mouse model.

To evaluate whether intestinal *E. coli* exacerbates pathological outcomes associated with cognitive impairments observed in 5×FAD mice, we measured hippocampal Aβ concentrations (Figures 5F and 5G). Where there were no differences in total Aβ (Figure 5F), the percentage of that Aβ that was found within the Triton and formic acid soluble fractions (representing Aβ in a more insoluble form) was significantly higher in the *E. coli-*treated mice than the vehicle controls (Figure 5G), suggesting an increase in amyloid aggregation but no change in overall production. This is further supported by qPCR data from the cortex of these animals demonstrating no change in murine *App* or *Bace1* transcripts based on colonization state (Figures S6A and S6B). Together, these data suggest that *E. coli* exposure exacerbates insoluble amyloid deposition without impacting amyloid precursor protein (APP) production, cleavage, or overall Aβ levels within the brain. Emphasizing the importance of this change in Aβ solubility, % insoluble Aβ was negatively correlated with performance on the object location test (*R*^2^ = 0.33), suggesting that changes in Aβ may be driving cognitive impairment in these animals (Figure S6C). However, there was no significant correlation between Aβ solubility and performance on the Barnes maze test, perhaps because so many of the *E. coli-*treated 5×FAD mice did not find the escape platform within the allotted 90-s period (Figure S6D). Profiling of cytokines and chemokines within the serum and hippocampus showed no differences in these inflammatory mediators following *E. coli* treatment (Figure S6E). In addition, expression of tight junction protein genes *Cldn2* and *Ocln* was unchanged in the cortex, suggesting no overt changes to blood-brain barrier permeability (Figures S6F and S6G). Similarly, in the intestine, no differences in cytokine/chemokine levels or expression of tight junction protein genes *Cldn5*, *Ocln*, and *Tjp1* were observed, suggesting that *E. coli* does not induce robust changes in intestinal inflammation or permeability (Figures S6H–S6K).

**Figure 6.**
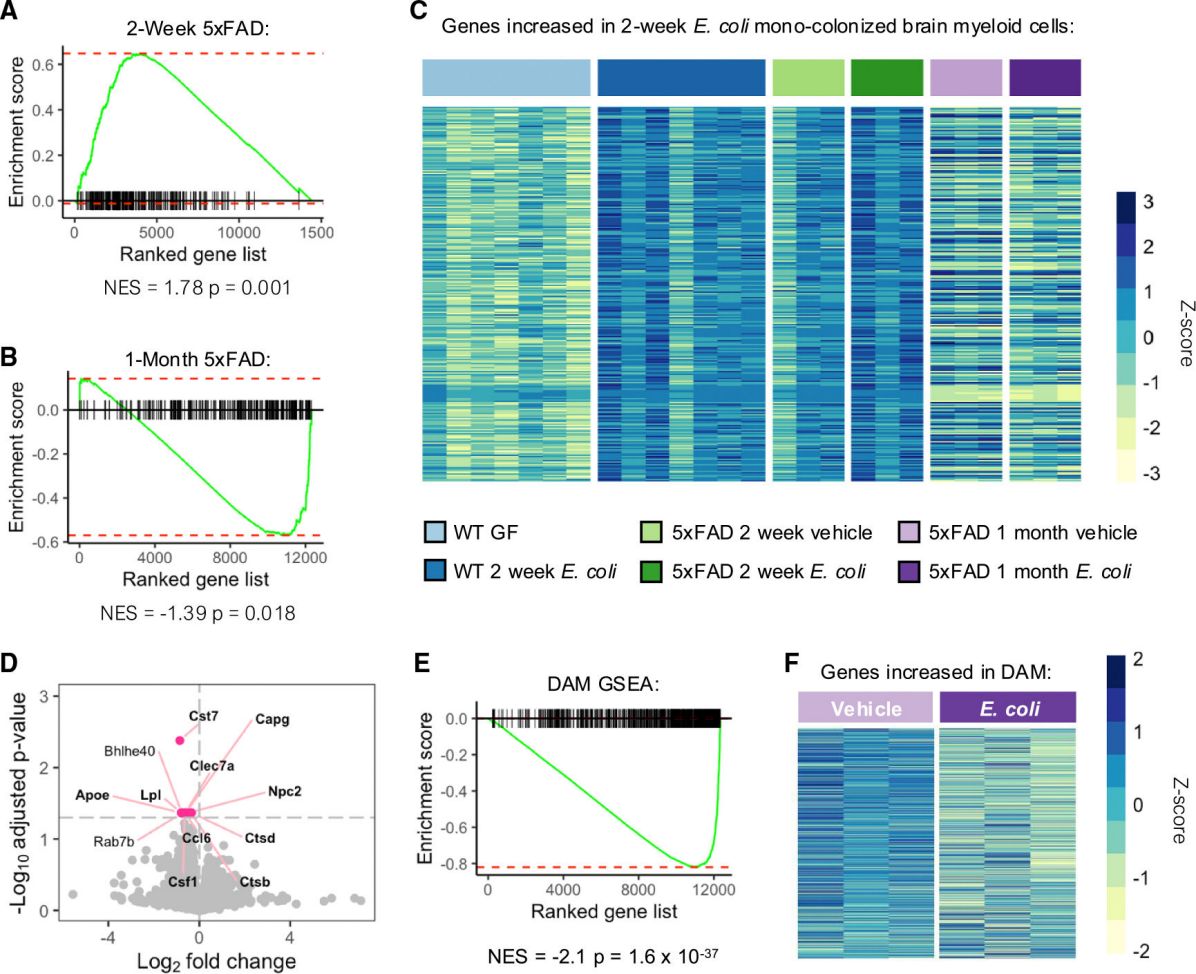
*E. coli* inhibits myeloid cell transition to the DAM activation state Bulk RNA-seq was performed on CD11b^+^ brain myeloid cells from 5×FAD mice after both 2 weeks and 1 month of treatment with either *E. coli* or vehicle control (Table S6; NIH GEO: GSE289590). (A and B) Gene set enrichment analysis (GSEA) was performed to determine whether the DEGs shown to be increased in *E. coli* mono-colonized mice at 2 weeks (DEGs; log fold change > |0.25|, *p* < 0.05; see Figure 2; Table S2) were similarly modulated in 5×FAD mice at both (A) 2 weeks and (B) 1 month. (C) The expression level (represented by *Z* score) of each of these mono-colonized DEGs is also shown in a heatmap. (D) Differential gene expression analysis was performed on the myeloid cells after 1 month of *E. coli* treatment. All DEGs (log fold change > |0|, *p* < 0.05) are labeled with the DEGs that are also known to be increased within the classical disease-associated microglia (DAMs) phenotype^40^ in bold. (E and F) GSEA of genes increased in DAM was performed with GSEA results in (E) and a heatmap of all DAM genes (represented by *Z* score) in (F). NES, normalized enrichment score. *n* = 7 for mono-colonized and GF mice and *n* = 3 for 5×FAD mice. In (C) and (F), rows represent individual mice.

We next sought to determine whether similar changes in brain myeloid cell transcription were occurring within *E. coli-*treated 5×FAD mice compared with wild-type *E. coli* mono-colonized mice. Bulk RNA-seq was performed on brain-derived CD11b^+^ cells from 5×FAD mice after both 2 weeks and 1 month of *E. coli* treatment (Figure 6; Table S6). At 2 weeks post-exposure, we did not detect any DEGs within 5×FAD mice, perhaps due to the smaller sample size. However, GSEA using the DEGs increased in 2-week *E. coli* mono-colonized (mixed by sex) mice demonstrated significant enrichment of the same gene set, emphasizing that a similar transcriptional response indeed occurred within both *E. coli* mono-colonized wild-type mice and *E. coli*-exposed, CONV 5×FAD mice (Figures 6A and 6C). By contrast, after 1 month of *E. coli* treatment, GSEA of DEGs from 2-week *E. coli* mono-colonized mice demonstrated a significant *negative* enrichment score, highlighting an inverse transcriptional state at this later time point (Figures 6B and 6C). We identified 12 DEGs within the CD11b^+^ cell population following 1 month of exposure, all of which were repressed (Figure 6D; Table S6). Despite the small number of DEGs, nearly all are highly relevant for AD. For example, *Apoe*—the greatest known genetic risk factor for late onset AD^49^ —had one of the largest log fold change values, and all but two of the DEGs (*Bhlhe40* and *Rab7b*) are known to be increased in the classical DAM phenotype.^40^ In support of this association, GSEA highlights a decrease in DAM genes following 1 month of *E. coli* exposure (Figures 6E and 6F). This suggests that *E. coli* exposure results in an inability for brain myeloid cells to transition into the initially protective DAM state and may potentially explain the worsened cognitive behaviors and pathology observed in *E. coli*-exposed 5×FAD mice. Overall, these results highlight how gut exposure to non-pathogenic *E. coli* alters brain-wide transcriptional state in healthy wild-type animals, impacts neuroimmune activation and functionality, and accelerates disease progression in a genetic model of AD.

## DISCUSSION

Increasing experimental evidence demonstrates that the gut microbiome maintains constant communication with the brain, shaping neurological function in both health and disease.^10,50^ We find that the gut microbiome shapes the transcriptional landscape of every major cell type in the brain, highlighting the breadth of microbiome-derived signaling. At a single-cell resolution, we identify both cell-type-specific and conserved transcriptional responses dependent on the presence of an intact microbiome. Further, our data delineate the shared and unique transcriptional responses elicited by particular gut microbial taxa, demonstrating the capacity for specific microbes to evoke distinct responses that are relevant for health and disease. For example, colonization with *E. coli* induces a broad transcriptional activation state associated with adaptive immune activation, including an increase in MHC class II antigen presentation and CD8^+^ T cell levels within the brain. Further, *E. coli* colonization modulates neurodegenerative disease pathways across varied brain cell types and exacerbates disease outcomes in a mouse model of AD. As a whole, this study highlights the association between the gut microbiome community, the active transcriptional landscape in the brain, and neurological disease susceptibility.

Over the past several decades, numerous studies have underscored the importance of the gut microbiome in shaping neurological function, including consequences for a wide range of neurological cell types.^10, 50^ In line with this, we observe a consistent pattern—shared across cell types—of microbiome-dependent influences on developmental and cellular organization processes within the hippocampus that are not typically found within adult mouse brains. Previous studies have demonstrated that microbiome-derived signals are necessary for appropriate neuroimmune development, with GF mice displaying an immature microglia phenotype and an inability to mount a typical inflammatory response.^7–9^ Our data further demonstrate a global dysregulation of genes associated with adaptive immune processes in the brain in the absence of a microbiome, emphasizing the importance of the microbiome for neuroimmune function. Even within healthy, wild-type mice, both excitatory and inhibitory hippocampal neurons display dysregulated transcription of genes assigned to neurodegenerative disease pathways in the absence of native microbiome-derived signals. These findings support the emerging role of the microbiome in modulating neurodegenerative disease outcomes, including numerous observations of microbiome-dependent pathology in both genetic and toxicant-induced models of neurodegenerative disease.^12,13^

Our study highlights the need to not only understand the consequences of the microbiome as a whole but also to pinpoint the specific effects of individual gut microbes on neurological functions. The composition of the gut microbiome differs across individuals, including significant differences in those living with neurological conditions compared with those who are neurologically healthy.^4^ In Parkinson’s disease (PD) for instance, the gut microbiome is well established to harbor particular, shared compositional features, even among diverse studies.^51^ In AD, however, human microbiome data are emerging, and such shared features across replicative studies have not yet appeared.^52^ Qualitatively, some microbes that are more consistently altered in those with AD include members of the *Bacteroidaceae* and *Enterobacteriaceae* families.^36–39,53–60^ These are diverse families, with both beneficial and pathogenic species, which each likely modulate host physiology differently based on their own unique genetic repertoire, the surrounding microbiome community structure, host genetics, age-related physiologies, and environmental/dietary input. We appreciate that our study did not test outcomes in wholly recolonized ex-GF mice, which would explore how a more intact, complex microbial community modifies neurophysiology, nor did we assess impacts of community colonization at distinct timepoints during development/aging. Nonetheless, our reductionist approach allows one to determine the sufficiency of individual species to induce specific neurophysiological changes. This allows for deeper insights into whether and how particular microbial associations may ultimately contribute to neurological outcomes, which can then be further tested in the context of a complex community or specific disease, as we did herein.

Through this reductionist mono-colonization approach, we found organism-specific effects on transcriptional activation of neuroimmune cells. This included one organism, *L. johnsonii*, whose colonization induced no transcriptional modulation compared with GF controls. This finding demonstrates that brain myeloid cells do not simply respond broadly and non-specifically to microbial colonization, but that there is indeed specificity in these interactions arising from the gut environment. Three other organisms, *B. thetaiotaomicron*, *C. celatum*, and *E. coli*, each induced unique transcriptional responses, demonstrating the specific neuroimmune modulatory capacity of these GI resident bacterial taxa. *E. coli* notably triggered an increase in expression of genes involved in antigen presentation and adaptive immune activation, pathways that were robustly decreased with colonization by both *B. thetaiotaomicron* and *C. celatum*. Similarly, colonization with *E. coli* alone induced expression of several pathways associated broadly with neurodegenerative diseases, including prion disease, AD, and ALS, within brain myeloid cells, emphasizing the disease-modulatory potential of this bacterium. The contrasting outcomes between colonization by these organisms raise important questions as to the signals derived from these species that trigger neurological responses. Both *B. thetaiotaomicron* and *E. coli* produce immune stimulatory LPS, which is often pointed to as a primary microbiome-derived contributor to neuroinflammation. However, these organisms elicit very different transcriptional responses, and neither induce genes involved in the canonical response to the LPS pathway (GO:0032496), suggesting that LPS signaling may not strongly contribute to the phenotypes we observe. These organisms also have differing capacities for short-chain fatty acid (SCFA) fermentation and bile acid metabolism, which may not only modulate peripheral immune responses but also have impacts within the CNS, including neuroimmune modulation and amyloid pathology.^28,61–63^ The strain of *E. coli* used in this dataset also produces the bacterial amyloid, curli, with both amyloidogenic and immune stimulatory properties.^64^ Our study herein provides a foundation and rationale to begin testing these comparative pathways across related organisms. For instance, using isogenic bacterial mutants, or animal models lacking specific receptors, would delineate the respective contributions of potential neuro-stimulatory microbial metabolites.

Microbiome-dependent transcriptional responses in the brain are dynamic. Our temporal analysis identified that immune cells and myelinating oligodendrocytes are acutely responsive to *E. coli* colonization, compared with neuronal populations, suggesting that these cell types may be particularly susceptible to microbiome-derived signals. Specifically, within microglia, expression of genes involved in adaptive immune pathways was initially robustly upregulated following colonization, but this response subsided by 4 weeks post-colonization. However, an increase in immune pathways, particularly those involved in adaptive immunity, subsequently became apparent in nearly every other cell type by 4 weeks post-colonization, suggesting that microglia are a focal point in relaying microbiome-derived signals to other cells in the brain. Functionally, neuroinflammatory transcriptional responses were associated with increased MHC class II production, T cell infiltration, and shifts in phagocytic capacity of brain-resident myeloid cells. This solidifies prior observations of microbiome-dependent shifts in microglia state during both development and in models of neurodegenerative disease,^7–9,20,65^ as well as recent reports of microbial modulation of T cell populations within the brain.^25–28^ It further suggests that microbiome-elicited microglia responses may trigger subsequent transcriptional impacts across other cell types in the brain. We show that microglia responses and subsequent neuronal transcriptional states are associated with the infiltration of CD8^+^ T cells. While the targets of these cells are not established in our model, CD8^+^ T cells are thought to play pathogenic roles in many neurodegenerative diseases.^41–45,66^ It may be that colonization with particular microbes promotes a CNS tropism for lymphocytes by first signaling to myeloid cells within the brain, which we observe as being robustly and acutely responsive to colonization. Future experiments using gnotobiotic mice lacking particular immune cells or receptors would clarify whether microglia or infiltrating T cells ultimately promote disease-relevant signaling to the rest of the neural cell population.

In support of a pathogenic contribution of *E. coli* to disease susceptibility, colonization with *E. coli* was sufficient to induce a transcriptional response associated with neurodegenerative diseases in wild-type mice, in the absence of disease-permissive genetics. Further, we demonstrated that increased exposure to *E. coli* within the 5xFAD mouse model of AD was sufficient to accelerate the development of cognitive decline and amyloid pathology. Gene expression patterns of brain-resident myeloid cells in *E. coli*-exposed 5xFAD mice mirrored *E. coli* mono-colonized mice at 2 weeks of exposure or colonization, expanding findings in gnotobiotic mice to a more naturalistic context. After 4 weeks of *E. coli* exposure, microglia displayed a relative decrease in classical DAM phenotypes (by both gene expression and CD11c positivity) in both wild-type and 5xFAD mice. As DAMs are thought to be an initially protective state that limits the development and progression of AD outcomes,^40,67^ this lack of protective response may explain why disease outcomes progressed more rapidly in *E. coli*-exposed 5xFAD mice. This *E. coli*-mediated exacerbation of neurodegenerative disease outcomes does not appear to be specific to AD, as we and others have observed that *E. coli* and related taxa within the *Enterobacteriaceae* family are sufficient to exacerbate pathologies in other models of neurodegeneration.^31–34^ Our data herein expand on this observation, identifying specific brain transcriptional responses evoked by the presence of *E. coli* within the microbiome that may contribute to detrimental outcomes across a range of neurological contexts. This is of particular importance given observations that *Enterobacteriaceae* are enriched in the gut microbiome in individuals with various neurodegenerative diseases.^12^

While we utilized a model of AD-relevant pathologies, our data serve as a foundation to understand how microbiome-dependent transcriptional responses associated with specific microbial species can modulate neurological disease susceptibility even in the absence of disease-promoting transgenics. Microbial signals were shown to impact a wide range of neurodegenerative disease pathways in nearly all cell types, underscoring the potential for microbiome modulation in numerous neurodegenerative diseases. Further, microbiome-dependent influences across hippocampal cell types have implications for a wide range of diseases involving hippocampus-dependent behaviors, including both memory and mood. While this study examined the transcriptional landscape within the entire hippocampus, future work delineating the effects of the microbiome within its sub-regions could provide important insights into potential microbiome-mediated consequences in disorders that preferentially impact these particular anatomical sites.

Systemic immune responses vary greatly in response to individual species, even within the same genera,^68^ suggesting that delineating individual microbial contributions is essential. While *E. coli* exemplifies how non-pathogenic microbiome-derived signals shape the neurological transcriptional landscape, our data demonstrate that individual bacterial species—and perhaps bacterial strains—will induce differential outcomes. For example, we highlight the seemingly inverse consequences of *B. theta* on neuroimmune transcriptional state compared with *E. coli*, yet it has been demonstrated that exposure to a related species of Bacteroides, *Bacteroides fragilis*, is sufficient to exacerbate AD outcomes.^69–71^ While abundances of both *Escherichia* and *Bacteroides* are reported to be differentially altered in people living with AD,^36–39,53–60^ public studies of the AD-associated microbiome are currently limited. Understanding the species-specific associations in these human conditions and their experimental contributions to neurological functions remains an important gap in the field. As data on the specific bacterial strains that are altered in patients with neurological disease remains limited, this study relied on bacterial type strains that are representative of prevalent species within the human gut microbiome. While these type strains likely possess many of the same attributes as those resident to the human gut, in the absence of in-depth metagenomic studies and subsequent mechanistic testing of strain-specific genetic features, the strain specificity of our observations remains unclear. Similarly, while *E. coli* was found to induce a unique response compared with the other bacterial species in this study, it is quite possible that other bacteria could induce a similar effect through a shared or convergent bacterial mechanism. Indeed, *B. thetaiotaomicron* and *C. celatum* are highly evolutionarily divergent (e.g., Gram-negative vs. Gram-positive) and yet in our hands evoked similar transcriptional responses in brain myeloid cells. Future mechanistic studies are therefore required to truly understand the pathway of gut-to-brain communication and the potential clinical relevance. Together, our results highlight the specificity and dynamics of microbiome-derived signals on the transcriptional landscape of the brain, serving as a foundation for the continued study of how the indigenous microbiome shapes overall brain health and disease susceptibility.

## RESOURCE AVAILABILITY

### Lead contact

Further information and requests for resources should be directed to and will be fulfilled by the lead contact, Timothy Sampson (trsamps@emory.edu ).

### Materials availability

This study did not generate new materials.

### Data and code availability

- Transcriptomic data have been deposited into the NIH GEO database and are available at the following accession numbers GEO: GSE289589, GSE289591, and GSE289590 for hippocampal snRNA-seq, mono-colonized CD11b^+^ bulk RNA-seq, and 5xFAD CD11b^+^ bulk RNA-seq, respectively, and are publicly available as of the date of publication. Numerical behavior data are provided at Zenodo (DOI: 10.5281/zenodo.18405899). Accession numbers are listed in the key resources table.
- All original code has been deposited on GitHub at https://github.com/LisaBlackmer/Blackmer-Raynolds-et-al.-2026 (DOI: 10.5281/zenodo.16996392) and is publicly available as of the date of publication. DOIs are listed in the key resources table. Any additional information required to reanalyze the data reported in this paper is available from the lead contact upon request.
- All other data reported in this paper will be shared by the lead contact upon request.

## STAR★METHODS

### EXPERIMENTAL MODEL AND STUDY PARTICIPANT DETAILS

#### Animals

##### Gnotobiotics

Germ-free (GF), male and female DBA/2N mice were originally obtained from Taconic Biosciences (#DBA2; RRID: IMSR_TAC:DBA2) following embryonic rederivation and bred within the Emory Gnotobiotic Animal Core (EGAC) for at least 3 generations prior to use in this study. GF animals were co-housed (with 2–5 same sex and age matched cage mates) in sterile cages within Parkbio rigid isolators. Mice were provided sterile food (Teklad Autoclavable Diet, 2019s) and water *ad libitum*. Microbiological testing (by culture and qPCR) was performed on all autoclaved materials entering isolators (including food, water, and bedding) as well as monthly within the isolators themselves. Prior to sacrifice or mono-colonization, all mice were transferred to sterile, static housing in specific pathogen-free (SPF) vivarium. Mono-colonization was performed by oral gavage with ∼10^8^ colony forming units (CFUs) of bacteria of interest within a sterile, class II biological safety cabinet. Colonization status of all mono-colonized and GF mice was confirmed by fecal culture at the time of sacrifice when the mice were 12–15 weeks old.

##### Conventionally-reared mice

Conventionally-raised (CONV) male and female DBA/2J mice were originally obtained from Jackson Laboratory (#000671; RRID: IMSR_JAX:000671) and co-housed (2–5 per cage) in static housing with food (LabDiet: 5001) and water provided *ad libitum* within an SPF facility. Female and male 5xFAD mice, on a congenic C57BL/6J background (Jackson Labs, #034848; MMRRC_034848-JAX) were maintained by crossing with C57BL/6J wildtype mice (IMSR_JAX:000664). Mice were co-housed (2–5 same sex mice per cage) with mixed genotype littermates in sterile, microisolator cages, under a 12h light/dark cycle with *ad libitum* access to sterile food (Teklad Autoclavable Diet, 2019s) and drinking water. Genotypes were confirmed with the following vendor-approved primers and their PCR parameters: APP Forward 5^′^- AGGACTGACCACTCGACCAG-3^′^, APP Reverse 5^′^-CGGGGGTCTAGTTCTGCAT-3^′^; PS1 Forward 5^′^- AATAGAGAACGGCAGGAGCA-3^′^, PS1 Reverse 5^′^- GCCATGAGGGCACTAATCAT-3^′^. All animal husbandry and experiments were performed in accordance with AVMA guidelines and approved by the Institutional Animal Care and Use Committee of Emory University (PROTO201900056).

#### Bacteria

*Bacteroides thetaiotaomicron* str. VPI 5482 (ATCC 29148), *Clostridium celatum* str. VPI 8759-1 (ATCC 27791), and *Lactobacillus johnsonii* str. VPI 7960 (ATCC 33200) were obtained from the American Type Culture Collection (ATCC). *Escherichia coli* str. K12 MC4100 (a lab-derived K12 strain^72^ and a kind gift from Matthew Chapman (University of Michigan^31^) and *B. thetaiotaomicron* were grown aerobically at 37° C in tryptic soy broth (BD #211825) and brain heart infusion (BD #237500) supplemented with hemin and vitamin K, respectively. *C. celatum* and *L. johnsonii* were grown anaerobically at 37°C in Chopped Meat carbohydrate (Anaerobe Systems AS-811) and de Man-Rogosa Sharpe (BD 288130) broth respectively. Bacterial cultures were resuspended at ∼10^8^ CFU in sterile 50% glycerol and 5% sodium bicarbonate, plated to confirm monoculture, and stored at −80°C until use. Vehicle control consisted of sterile 50% glycerol (v/v) and 5% sodium bicarbonate (w/v) also stored at −80°C.

### METHOD DETAILS

#### Bacterial enrichment

Male and female, conventionally raised 2-month-old 5xFAD and wild-type littermates were treated with an antibiotic cocktail consisting of 1mg/mL neomycin, 1mg/mL ampicillin, and 5mg/mL vancomycin in sterile water for 1 week. After antibiotic treatment, mice were randomly assigned to receive either ∼10^8^ CFU of *E. coli* by oral gavage 3x per week or vehicle (sterile 50% glycerol and 5% sodium bicarbonate) gavage. While cages consisted of mixed genotype animals, each cage of mice only received a single treatment to prevent cross-contamination. Each week, the colonization status of the mice was monitored by fecal culture.

#### Behavioral testing

Roughly equal numbers of male and female 5xFAD (n = 11–15) and wildtype (n= 9–23) littermates underwent behavioral testing after 1 month of enrichment, at 3 months of age. All behavioral testing was performed during the animal’s light cycle. Before the start of any test, mice were habituated to the testing room in their home cage for 1 h. Oral gavage was continued 3x per week throughout the behavioral testing period to ensure continued *E. coli* exposure. Whenever possible, mice were gavaged after behavioral testing had been completed for the day. If this was not possible due to the large number of mice used in our study, mice were split into batches (with equal numbers of vehicle and *E. coli* mice) and half were gavaged at least 5 hours before testing began. All behavioral tracking and analysis were performed using EthoVision XT software (Noldus Information Technology, Wageningen, the Netherlands) and the testing arenas/objects were cleaned between trials with 70% ethanol to eliminate bacterial contamination and olfactory cues. Mice were tested on the following tests in order as described previously^73^ (see Figure 5A for a behavioral testing timeline). Behavior data are available at Zenodo (DOI: 10.5281/zenodo.18405899).

##### Open field test (OFT)

As measures of motor and anxiety-like behavior, mice were placed in a 45cm square open field box for 10 minutes and allowed to explore freely. Distance traveled and time spent in the center (20x20cm) was recorded. The OFT also served as habituation for the object location test (OLT).

##### Object location test (OLT)

Twenty four hours after the OFT, the OLT was run to assess short term spatial memory as described step-by-step in.^74^ The same open field box used in the OFT was used for the OLT, but additional landmarks (large papers with stripes, stars, or dots of different colors) were placed on 3 out of the four walls to allow for spatial orientation. During the initial study phase, mice were placed in the box with two identical copies of an object (either a plastic chess piece or 5mL Eppendorf tube) and allowed to explore freely for 10 minutes. The mice were then returned to their home cage for a 10-minute retention delay. During the testing phase, mice were returned to the box where one of the two objects had been moved to a novel location and allowed to explore freely for 5 minutes. Object exploration was considered time spent with the mouse’s nose within 2cm of an object. Novelty preference was measured by taking the percent of total object exploration time that was spent exploring the moved object. A novelty preference significantly above 50% chance levels is indicative of intact memory as mice generally seek out the moved object.

##### Y-maze

In order to evaluate spatial working memory capacity, the Y maze test was performed as described in detail in.^75^ Mice were placed in a plastic Y shaped maze and allowed to explore freely for 8 minutes while the order of entries into each arm of the maze was recorded. Percent alternation was calculated by taking total number of alternations (consecutive entries into 3 arms before repeating any arms) divided by the maximum possible alternations (total entries minus 2) and multiplying by 100. Mice with intact working memory display higher percent alternation as they prefer to explore previously unvisited areas.

##### Barnes maze

Longer term spatial learning and memory was evaluated using the Barnes maze test adapted from,^76^ and described in detailed in.^77^ Testing was performed over a 6-day period using a 92cm diameter, 20-hole, Barnes maze (MazeEngineers) with consistent extra-maze cues throughout. First, mice underwent a habituation trial in which they were placed on the maze with bright lights and white noise (66–70 dB) playing for 20 seconds then gently guided to an escape hole leading to a dark box filled with sterile bedding. After entering the escape box, the white noise was immediately turned off and the mice were allowed to acclimate to the escape box for 2 minutes. The mice then underwent 5 consecutive training trails spread over 2 days. During each training trial, mice were placed in the center of the maze (with white noise and bright lights on) and allowed to explore for up to 3 minutes. If the mice entered the escape box during this period, the noise was turned off and they were allowed to rest for 1 minute. Mice that did not enter the escape box after 3 minutes were gently guided to the proper hole before being allowed to rest. The probe trial occurred 72hr after completion of the final training trail. During the probe trial, the escape box was removed, and mice were placed on the maze (lights and noise on) to explore for 90 seconds. During both training and probe trials, primary latency (the time it took for the mouse to first check the escape hole) was recorded by hand.

##### Intestinal behaviors

To evaluate whether colonization disrupts gastrointestinal (GI) function, a subset of roughly equal male and female mono-colonized (n=6–15) and 5xFAD mice (n=5–9) underwent a set of GI tests. First, colonic transit was measured by fecal output testing described in detail in.^78^ Fecal output testing was performed within a level II biosafety cabinet within the animal’s vivarium with no prior habituation period. Mice were placed in individual sterile 1L plastic beakers for 30 minutes and the number of fecal pellets produced was recorded every 5 minutes. Fecal water content was measured by collecting the fecal pellets produced during the fecal output test and weighing them before and after drying at 100°C for 48hr. As a measure of total intestinal transit time, carmine red dye elution was performed as described in.^79^ Testing was performed in the behavioral testing room. Mice were allowed to acclimate for 1 h before undergoing oral gavage with 100μl of sterile carmine red dye (6% w/v) (Sigma, C1022) dissolved in 0.5% methylcellulose (w/v; Sigma, M7027). Mice remained in their home cages for 2 hours then were transferred to individual sterile cages devoid of bedding, but with access to a small amount of sterile food and water. The cages were observed every 15 minutes for the presence of a red fecal pellet. Once a red pellet was discovered, the time was recorded, and the mouse was returned to its home cage.

##### Tissue collection and processing

Mice were humanely euthanized via open-drop isoflurane overdose followed by cardiac puncture to collect blood samples, perfusion with PBS, and exsanguination. To collect serum, blood was immediately placed into vacuette collection tubes (Griener #454243) and spun at room temperature at 1,800 x *g* for 10 minutes. Serum was then stored at −80°C until use. Brain tissue was either: A) immediately processed by immunopanning, magnetic activated cell sorting (MACS) or flow cytometry (see below); B) dissected and flash frozen in liquid nitrogen and stored at −80° C for later protein, nuclei, or RNA isolation; or C) fixed for 24hr in 4% paraformaldehyde and stored at 4° C in 0.01% sodium azide for immunohistochemistry. Intestinal tissue was removed from the mouse and colon length was measured as a marker of overall intestinal inflammation. Approximately 1cm of tissue was dissected from the ilium and proximal colon, flash frozen in liquid nitrogen and stored at −80° C for multiplex ELISA and qPCR.

##### Brain myeloid cell enrichment and flow cytometry

Immediately after sacrifice, brains (the left hemisphere was used for immunopanning or magnetic activated cell sorting (MACS) where the entire brain was used for flow cytometry) were homogenized using a Wheaton dounce tissue grinder (with 0.114 ±0.025mm pestle clearance) in culture media comprised of HBBS with 1.5% HEPES, and 0.5% glucose. Cells were then transferred to a 35% isotonic percoll gradient solution and spun 20 minutes at 700 x *g* to remove the myelin layer. For immunopanning and MACS (but not flow cytometry), transcription (Actinomycin D, Sigma #A1410) and translation (Anisomycin, Sigma #A9789) inhibitors were added to every solution throughout both procedures to prevent transcriptional changes that could impact downstream results. The remaining cells were washed several times in HBBS culture media before undergoing immunopanning, MACS, or flow cytometry.

##### Immunopanning

Cells were resuspended in 0.02% BSA in PBS and incubated at room temperature on a 10cm petri dish with anti-CD11b and secondary antibody already adhered to the dish (Thermo Scientific, 14-0112-82 and Jackson 112-005-167) for 10–15 minutes. After incubation the unadhered cells were washed away and remaining cells scraped off the plate directly into Trizol (Zymo Research R20501-200) and stored at −80°C until later use.

##### MACS

Sorting was performed according to the manufacturers (Miltenyi Biotec) guidelines. Samples were resuspended in PB buffer (0.5% BSA in PBS pH 7.2) with CD11b MicroBeads (Miltenyi Biotec # 130-093-634) and incubated for 15 minutes at 4°C protected from light. Cells were then washed and resuspended in PB buffer before being placed on an LS column (Miltenyi Biotec #130-042-401). Magnetic separation was repeated on a second LS column to maximize purity. After separation, cells were resuspended in Trizol and stored at −80°C for later use.

##### Flow Cytometry

For the *ex vivo* phagocytosis assay 20μM HiLyte^™^ Fluor 647-labeled beta-Amyloid (1–42) (AS-64161) diluted in 1% ammonium hydroxide was first incubated at room temperature for six days to allow for fibrilization. Brain homogenates were then resuspended in 12.5μL fibrilized HiLyte^™^ Fluor 647-labeled beta-Amyloid (1–42) and incubated at 37°C for 30 minutes to allow for amyloid uptake. Cells were then washed with 1mL of cold PBS and blocked with anti-Fc receptor (1:40, cat #14-0161-81) for 15 minutes on ice. Cells were resuspended in an antibody cocktail consisting of antibodies against CD45 (1:320, Clone 30-F11, eBioscience cat #364-0451-80), CD11b (1:320, Clone M1/70, eBioscience cat #365-0112-82), CD3e (1:80, Clone 145-2C11, eBioscience cat# 376-0031-82), CD8a (1:160, Clone 53-6.7, eBioscience cat# 368-0081-82), CD4 (1:320, Clone GK1.5, eBioscience cat# 53-0041-82), CD38 (1:320, Clone 90, eBioscience cat# 61-0381-82), CD11c (1:160, Clone N418, eBioscience cat #25-0114-82), and Ly6C (1:320, Clone HK1.4, eBioscience cat# 48-5932-82) and incubated on ice for 30 minutes, shaken periodically. A fixable viability dye was also used according to the manufacturer’s instructions (1:4000, LIVE/DEAD FIX NIR cat# L34975). After antibody staining, cells were washed with FACS buffer (2.5% 1M HEPES, 0.2% 0.5M EDTA, and 1% FBS in PBS). Antibody controls were made using one drop of UltraCompensation Plus Beads (Thermo Fisher cat #01-3333-42) and individual antibodies at the same concentrations listed above. Beads and antibodies were incubated for 30 minutes on ice in the dark and then diluted with 1mL of FACS buffer. The diluted beads were spun down at 500 x *g* for 5 min, the supernatant was removed, and the remainder was resuspended in 200μL of FACS buffer.

Blood was collected into EDTA tubes and diluted 1:4 in FACS buffer (2.5 % 1M HEPES, 0.2% 0.5M EDTA, and 1% FBS in PBS). Cells were blocked with anti-Fc receptor (1:40, cat #14-0161-81) for 15 minutes on ice. Cells were resuspended in an antibody cocktail consisting of antibodies against CD45 (1:320, Clone 30-F11, eBioscience cat #364-0451-80), CD11b (1:320, Clone M1/70, eBioscience cat #365-0112-82), CD3e (1:80, Clone 145-2C11, eBioscience cat# 376-0031-82), CD8a (1:160, Clone 53-6.7, eBioscience cat# 368-0081-82), CD4 (1:320, Clone GK1.5, eBioscience cat# 53-0041-82), CD38 (1:320, Clone 90, eBioscience cat# 61-0381-82), CD11c (1:160, Clone N418, eBioscience cat #25-0114-82), and Ly6C (1:320, Clone HK1.4, eBioscience cat# 48-5932-82) and incubated on ice for 30 minutes, shaken sporadically. A fixable viability dye was also used according to the manufacturer’s instructions (1:4000, LIVE/DEAD FIX NIR cat# L34975). Cells were washed with FACS buffer. Red blood cells were lysed by incubating at room temperature for 15 minutes in 800μl of 1x lysing buffer (BD Cat #349202). Cells were washed with FACS buffer twice, then resuspended in 400μl of 2% PFA and incubated in the dark at room temperature for 30 minutes.

Brain and blood samples, along with the proper controls were sorted using a Cytek Cell Sorter at the Emory School of Medicine Flow Cytometry Core. Data was analyzed in Flowjo Version 10.10.0.

#### Single nuclei preparation and sequencing

Flash frozen hippocampal samples from 12–15-week-old female GF, conventionally raised, and mono-colonized mice (n = 4) underwent a nuclei isolation protocol adapted from.^80^ Frozen tissue was placed in homogenization buffer (consisting of 0.26M sucrose, 0.03M KCl, 0.01M MgCl_2_, 0.02M Tricine-KOH pH 7.8, 0.001M DTT, 0.5mM Spermidine, 0.05mM Spermine, 0.3% NP40, and protease inhibitor) and homogenized using both pestle A (0.0030–0.0050 in. clearance) and pestle B (0.0005–0.0025 in. clearance) of a KIMBLE dounce tissue grinder (Sigma D8938). Density gradient centrifugation (3,000 x *g* for 20 min at 4°C) was performed using iodixanol and the nuclei band was captured at the 30%-40% iodixanol interface. Nuclei were washed in RSB buffer (0.01M Tris-HCL pH 7.5, 0.01M NaCl, 0.003M MgCl_2_, and 0.1% Tween-20) then samples were combined such that each treatment group was represented by two samples, each containing roughly equal numbers of cells from two mice within a single treatment group. Then, samples immediately underwent cell capture using the Chromium Next GEM Single Cell 3’ Kit v3.1 (10x Genomics # PN-1000268) according to the manufacturer’s guidelines. Cells were loaded onto a Chromium Next GEM Chip and run using a Chromium Controller and library preparation steps were performed according to the manufacturer’s guidelines. Pooled samples were sequenced on a NovaSeq X Plus 25B 2x150 for 5-6.25B PE reads total by Admera Health (South Plainfield, NJ). Raw sequencing files are available at NIH GEO: GSE289589.

#### Single nucleus RNA-seq analysis

Reads were aligned to the mouse reference genome (GRCm39) using the Cell Ranger pipeline (version 4.0.10, 10x Genomics). The Cellbender^81^ (version 0.3.0) remove-background function was used to minimize the effects of ambient RNA (expected cells=5,500 and total droplets included=12,000). The snRNA-seq libraries were imported into R (version 4.3.2) using Seurat^82^ (version 5.1.0) then filtered to only include cells with 200–7,500 features and less than 6% mitochondrial reads. Data was normalized, scaled, and clustered using Seurat defaults and doublets were removed using DoubletFinder^83^ (version 2.0.4) with a multiplex rate of 0.8% per 1,000 cells. Samples where integrated using Seurat RPCA Integration then re-clustered to create an integrated UMAP. Seurat “FindAllMarkers” function was used (min.pct = 0.25, thresh.use = 0.25) to identify marker genes for each cluster. Manual cell type annotation was performed by identifying cell types with high marker gene expression within the Allen Brain Cell Atlas.^23^ Differential expression analysis was performed within each cell cluster of interest by using MAST within the Seurat “FindMarkers” function. Genes were considered differentially expressed if they had an absolute log fold change value greater than 1 and adjusted p value of less than 0.001. Functional annotation clustering was performed using Metascape^84^ (version 3.5.20240901) with all DEGs except genes without a canonical name (those ending in “rik” or beginning with “Gm”) and GSEA was run using clusterProfiler^85^ (version 4.10.1). All analysis code is availible on GitHub and deposited to Zenodo (DOI: 10.5281/zenodo.16996392).

#### Bulk RNA-seq preparation and sequencing

RNA was extracted from isolated brain myeloid cells using the Qiagen RNeasy Kit (#74106) according to the manufacturer’s guidelines. Bulk RNA-sequencing libraries were created for each sample using the Takara SMART-Seq mRNA HT LP kit (#634792) according to the manufacturer’s protocol and recommendations. Sequencing was performed by Admera Health (South Plainfield, NJ) using a NovaSeq or HiSeq to achieve approximately 85 million total reads per sample. Raw sequencing files are available at NIH GEO: GSE289591, GSE289590 for mono-colonized 5xFAD samples, respectively.

#### Bulk RNA-seq analysis

Sequencing quality control was performed using FastQC (version 0.11.9) and reads were pseudoaligned to the mouse reference genome (GRCm39) using Kallisto (version 0.44.0).^86^ Files were imported into R (version 4.3.2) and counts were converted into TMM normalized log_2_ counts per million. Genes expressed in less than 3 samples were excluded from the analysis. Differential gene expression analysis was performed using limma^87^ (version 3.58.1) and edgeR^88^ (version 4.0.16). Differentially expressed genes were filtered to only include genes enriched in microglia compared to other brain cell types based on expression levels published by the Brain RNA-seq Website.^89^ Genes were considered differentially expressed if they had a Bonferroni adjusted p value less than 0.05. Functional annotation clustering was performed using DAVID^90,91^ and clusterProfiler^85^ (version 4.10.1), gene set enrichment analysis was performed by fgsea^92^ (version 1.28.0). All analysis code is availible on GitHub and deposited to Zenodo (DOI: 10.5281/zenodo.16996392).

#### Real-time quantitative PCR

RNA was extracted from flash-frozen tissue using the Qiagen RNeasy Mini Kit (cat#: 74106) and concentrations for each sample were measured using a NanoDrop. RNA was converted to cDNA using the iScript cDNA Synthesis Kit (Bio-Rad, cat#: 1708891) following the manufacturer’s instructions. cDNA was diluted to reach a final amount of 10ng cDNA per reaction. Primer pairs, listed in the key resources table, were diluted from 100μM stocks to 10μM combined working stocks. Per sample and gene of interest, a 12μl reaction mix was created: 6μl SYBR Green PCR Master Mix (Thermo Fisher Scientific, Cat#: 4309155), 2μl diluted primer pairs, 2μl deionized water, and 2μl diluted cDNA. Each sample was tested in technical duplicate, and appropriate controls and blanks were used (no-template control, cDNA only, deionized water only). The reactions were performed on an Applied Biosystems 7900HT Fast Real-Time PCR System.

#### Protein extraction and ELISA

Protein was extracted from flash frozen brain and gut samples by sonication in homogenization buffer consisting of 125mM Tris, 15mM MgCl_2_, 2.5mM EDTA (PH 7.2), 1% Triton X 100, and protease inhibitor (Roche #11697498001, 1 tablet per 10mL). Samples were centrifuged at 20,800 x *g* for 10 minutes at 4°C. The protein concentration of the supernatant was measured using a Pierce BCA Protein Assay Kit (Thermo #23225) according to the manufacture’s guidelines. When quantifying amyloid beta levels, a three part protein solubility protocol outlined in^93^ was performed instead. First, the tris soluble fraction was isolated by sonication in 125mM Tris, 15mM MgCl_2_, 2.5mM EDTA (PH 7.2), and protease inhibitor (Roche #11697498001, 1 tablet per 10mL). Following centrifugation, the pellet was then re-sonicated in the above buffer with the addition of 1% Triton X 100 to isolate the triton soluble fraction. Finally, the remaining pellet was sonicated in buffer containing 70% formic acid to extract the most insoluble protein fraction. Protein samples as well as pure serum samples were then run using multiplex ELISA (Meso Scale Discovery V-PLEX Proinflammatory Panel 1 (mouse) Kit # K15048D and V-PLEX human amyloid beta peptide kit #K15200E).

#### Immunohistochemistry

Paraformaldehyde fixed brain hemispheres were transferred to a 30% sucrose solution for 24–48 h then frozen in Tissue-Tek O.C.T. compound (#4583) and sliced into 40μm coronal sections using a cryostat. Two to four sections representing the anterior hippocampus including the third and lateral ventricles were stained per mouse. Antigen retrieval was performed prior to IBA1 staining by heating the tissue to 90°C for 5 minutes in a sodium citrate antigen retrieval buffer (10 mM tri-sodium citrate dihydrate and 0.43mM Tween 20, pH 6.0). Tissue was then blocked in 1% BSA, incubated in anti-IBA1 antibody (Wako #019-19741 1:1,000), and anti-rabbit 594 secondary (Thermo #A11012, 1:1,000). Imaging was performed using a Keyence BZ-X Series microscope (Itasca, IL) at 20X magnification. Images were processed in Fiji using a macro created by the Emory Integrated Cellular Imaging Core that auto thresholded the images using “RenyiEntropy dark,” converted to mask, and calculated IBA1% area within a hand traced region of interest around the hippocampus. MHCII, CD31, and CD206 staining was performed without antigen retrieval using the following antibodies and concentrations: MHCII (BioLegend #107602) 1:300 with anti-rat biotin (Thermo #A187843) and streptavidin 488 (Thermo #S31354); CD31(R&D Systems # AF3628) 1:400 with anti-rabbit 594 (Thermo #A21207) 1:1,000; and CD206 (Cell Signaling # 24595T) 1:400 with anti-goat 647 (Thermo #A21447). A streptavidin/biotin blocking kit (Vector labs SP-2002) was used in conjunction with biotinylated antibodies to improve MHCII signal. Images were taken at 20x using a Leica SP8 multiphoton microscope and analyzed using Fiji “Li” and “Moments” auto thresholding for MHCII and CD206 respectively. Microscopy and image analysis was performed by a blinded lab member.

### QUANTIFICATION AND STATISTICAL ANALYSIS

#### Overview of Statistical Tests

Unless otherwise indicated in the figure legends, data are expressed as mean ± SEM. Sample sizes are indicated in the figure legends, and when possible, each individual sample is represented as its own point on each graph. In all analyses except snRNA-seq, a sample represents an individual mouse. In SnRNA-seq a single sample represents combined data from two mice of the same treatment. Statistical tests for all non-transcriptomic data were performed using GraphPad Prism 8. One-way ANOVAs were used to compare mono-colonized mice and T tests were used to compare enriched (5xFAD and wildtype) mice. The object location test was analyzed by a one sample T test comparing each treatment to the 50% chance level. Unless otherwise noted significance was determined to be a *p* value of less than 0.05. Details on transcriptomic data analysis can be found in their respective sections above. All unique code is publicly available on GitHub and deposited to Zenodo (DOI: 10.5281/zenodo.16996392).

## Supplementary Material

7

6

5

4

3

2

1

Supplemental information can be found online at https://doi.org/10.1016/j.cels.2025.101481.

## Figures and Tables

**Table:** KEY RESOURCES TABLE

REAGENT or RESOURCE	SOURCE	IDENTIFIER
Antibodies		

Rat monoclonal anti-CD11b	Thermo Fisher Scientific	Cat# 14-0112-82; RRID:AB_467108
Rabbit polyclonal anti-IBA1	FUJIFILM Wako Pure Chemical Corporation	Cat# 019-19741; RRID:AB_839504
Rat monoclonal anti-MHCII	Biolegend	Cat# 107602; RRID:AB_313317
Goat polyclonal CD31/PECAM-1	R and D Systems	Cat# AF3628; RRID:AB_2161028
Rabbit monoclonal anti-CD206/MRC1	Cell Signaling Technology	Cat# 24595; RRID:AB_2892682
Goat anti-rat IgG	Jackson ImmunoResearch Labs	Cat# 112-005-167; RRID:AB_2338101
Goat anti-rabbit IgG 594	Thermo Fisher Scientific	Cat# A-11012; RRID:AB_2534079
Donkey anti-rat IgG Biotin	Thermo Fisher Scientific	Cat# A18743; RRID:AB_2535520
Streptavidin 488 Conjugate	Thermo Fisher Scientific	Cat# S32354; RRID:AB_2315383
Donkey anti-goat IgG 647	Thermo Fisher Scientific	Cat# A-21447; RRID:AB_141844
Donkey anti-rabbit IgG 594	Thermo Fisher Scientific	Cat# A-21207; RRID:AB_141637
Rat anti-CD4 (GK1.5) Alexa Flour 488	Thermo Fisher Scientific	Cat# 53-0041-82; RRID:AB_469893
American hamster anti-CD11C (N418) PE-Cyanine5.5	Thermo Fisher Scientific	Cat #25-0114-82; RRID:AB_469590
Rat anti-CD38 (90) Brilliant Violet 605	Thermo Fisher Scientific	Cat #61-0381-82; RRID:AB_2848484
Rat anti-CD8a (53-6.7) Brilliant Ultra Violet 805	Thermo Fisher Scientific	Cat# 368-0081-82; RRID: AB_2896078
American hamster anti-CD3e (152-2C11) Brilliant Ultra Violet 661	Thermo Fisher Scientific	Cat# 376-0031-82; RRID:AB_3099348
Rat anti-CD11b (M1/70) Brilliant Ultra Violet 563	Thermo Fisher Scientific	Cat# 365-0112-82; RRID:AB_2925363
Rat anti-CD45 (30-F11) Brillant Ultra Violet 496	Thermo Fisher Scientific	Cat# 364-0451-80; RRID:AB_2925332
Rat anti-Ly6C (Hk1.4) eFluor 450	Thermo Fisher Scientific	Cat #48-5932-82; RRID:AB_10805519
Goat anti-rabbit IgG Alexa Fluor 647	Thermo Fisher Scientific	Cat# A21244; RRID: AB_2535812

Bacterial and virus strains		

*Bacteroides thetaiotaomicron* str. VPI 5482	American Type Culture Collection (ATCC)	Cat# 29148
*Clostridium celatum* str. VPI 8759-1	American Type Culture Collection (ATCC)	Cat# 27791
*Lactobacillus johnsonii* str. VPI 7960	American Type Culture Collection (ATCC)	Cat# 33200
*Escherichia coli* str. K12 MC4100	U. Michigan (M. Chapman)	NCBI Taxon: 1403831

Chemicals, peptides, and recombinant proteins		

Tryptic soy broth	BD	Cat# 211825
Brain heart infusion broth	BD	Cat# 237500
Chopped meat carbohydrate broth	Anaerobe Systems	Cat# AS-811
de Man-Rogosa Sharpe broth	BD	Cat# 228130
Neomycin sulfate	Thermo Fisher Scientific	Cat# BP266925
Ampicillin sodium salt	Thermo Fisher Scientific	Cat# BP1760-25
Vancomycin hydrochloride	Thermo Fisher Scientific	Cat# AAJ6279006
Carmine powder	Sigma-Aldrich	Cat# C1022
Methyl cellulose	Sigma-Aldrich	Cat# M7027
Actinomycin D from *Streptomyces sp*.	Sigma-Aldrich	Cat# A1410
Anisomycin from *Streptomyces griseolus*	Sigma-Aldrich	Cat# A9789
cOmplete protease inhibitor cocktail	Roche	Cat# 11697498001
LIVE/DEAD Fixable Near-IR Dead Cell Stain Kit for 633 or 635nm excitation	Thermo Fisher Scientific	Cat# L34975
beta-Amyloid (1-42), HiLyte^™^ Fluor 647-labeled - 0.1 mg	Anaspec	Cat# AS-64161
Brilliant Stain Buffer	Thermo Fisher	Cat #00-4409-42
UltraCompensation Plus Beads	Thermo Fisher	Cat# 01-3333-42
FACS Lysing Solution	BD	Cat# 349202

Primers		

*APP* (human for 5xFAD genotype validation)	IDT DNA	Fwd 5′ - AGGACTGACCACT CGACCAG-3′, APP Reverse 5’-CGG GGGTCTAGTTCTGCAT-3′
*PS1* (human for 5xFAD genotype validation)	IDT DNA	Fwd 5′- AATAGAGAACGG CAGGAGCA-3′, PS1 Reverse 5′- GCCATGAGGGCACT AATCAT-3
*App* qPCR	IDT DNA	Fwd: 5’-GGTGCTGAGTCATCAA GCATAC-3’ Rev: 5’-GAACTTCA ACGTAGGTTGGGAA-3’
*Bace 1* qPCR	IDT DNA	Fwd: 5’-CAGTGGGACCACCA ACCTTC-3’ Rev: 5’-GCTGCCTT GATGGACTTGAC-3’
*Cldn5* qPCR	IDT DNA	Fwd: 5’-AGATTTCATCGGTGA AGTAGG-3’ Rev: 5’-GGACATTA AGGCAGCATCTA-3’
*Gapdh* qPCR	IDT DNA	Fwd: 5’-TGGCCTTCCGTGT TCCTA-3’ Rev: 5’-GAGTTGCT GTTGAAGTCGCA-3’
*Ocln* qPCR	IDT DNA	Fwd: 5’-TGGAAAGTCCACCT CCTTACAGA-3’ Rev: 5’-CCGG ATAAAAAGAGTACGCTGG-3’
*Tjp1* qPCR	IDT DNA	Fwd: 5’-CCTGAAGGAATTGAG CAAGA-3’ Rev: 5’-GCAGAGTT TCACCTTTCTCT-3’

Critical commercial assays		

Chromium Next GEM Single Cell 3′ Kit v3.1	10x Genomics	Cat# PN-1000268
RNeasy Kit	QIAGEN	Cat# 74106
SMART-Seq mRNA HT LP kit	Takara Bio	Cat# 634792
Pierce BCA Protein Assay Kit	Thermo Fisher Scientific	Cat# 23225
V-PLEX Proinflammatory Panel 1 (mouse) Kit	Meso Scale Discovery	Cat# K15048D
V-PLEX human amyloid beta peptide kit	Meso Scale Discovery	Cat# K15200E
Streptavidin/Biotin Blocking Kit	Vector Laboratories	Cat# SP-2002

Deposited data		

Raw and analyzed data	This paper	NCBI GEO: GSE289589, GSE289590, and GSE289591
Mouse reference genome NCBI assembly GRCm39	Genome Reference Consortium	https://www.ncbi.nlm.nih.gov/datasets/genome/GCF_000001635.27/
Allen Brain Cell Atlas	Allen Institute for Brain Science	https://portal.brain-map.org/atlases-and-data/bkp/abc-atlas
Brain RNA-seq	10.1523/jneurosci.1860-14.2014	https://brainrnaseq.org/

Experimental models: Organisms/strains		

Mouse: DBA2/NTac	Taconic Biosciences	RRID:IMSR_TAC:DBA2
Mouse: DBA2/J	Jackson Laboratory	Cat# 000671; RRID: IMSR_JAX:000671
Mouse: 5xFAD	Jackson Laboratory	Cat# 034848; RRID:MMRRC_034848-JAX

Software and algorithms		

EthoVision XT (version 16.0)	Noldus Information Technology	https://noldus.com/ethovision-xt RRID:SCR_000441
Fiji (version 2.14.0/1.54f)	10.1038/nmeth.2019	https://imagej.net/software/fiji/#downloads RRID:SCR_002285
GraphPad Prism (version 10.2.3)	Dotmatics	https://www.graphpad.com/ RRID:SCR_002798
FlowJo (Version 10.10.0)	Becton, Dickinson & Company (BD)	RRID:SCR_008520
Code for single cell and bulk RNA-seq analysis	This Paper	10.5281/zenodo.16996392
Cell Ranger (version 4.0.10)	10x Genomics	https://github.com/10XGenomics/cellranger RRID:SCR_017344
Cellbender (version 0.30)	10.1038/s41592-023-01943-7	https://cellbender.readthedocs.io/en/latest/installation/index.html RRID:SCR_025990
R (version 4.3.2)	The R Foundation	https://www.r-project.org/ RRID:SCR_001905
RStudio (version 2023.12.1+402)	Posit Software, PBC	https://posit.co/download/rstudiodesktop/ RRID:SCR_000432
Seurat (version 5.1.0)	10.1038/s41587-023-01767-y	https://satijalab.org/seurat/ RRID:SCR_007322
DoubletFinder (version 2.04)	10.1016/j.cels.2019.03.003	https://github.com/chris-mcginnis-ucsf/DoubletFinder RRID:SCR_018771
Metascape (version 3.5.20240901)	10.1038/s41467-019-09234-6	https://metascape.org/gp/index.html#/main/step1 RRID:SCR_016620
FastQC (version 0.11.9)	Babraham Bioinformatics	https://www.bioinformatics.babraham.ac.uk/projects/fastqc/ RRID:SCR_014583
Kallisto (version 0.44.0)	10.1038/nbt.3519	https://github.com/pachterlab/kallisto RRID:SCR_016582
Limma (version 3.58.1)	10.1093/nar/gkv007	https://bioconductor.org/packages/release/bioc/html/limma.html RRID:SCR_010943
EdgeR (version 4.0.16	10.1093/bioinformatics/btp616	https://bioconductor.org/packages/release/bioc/html/edgeR.html RRID:SCR_012802
DAVID Bioinformatics	10.1093/nar/gkac194 and 10.1038/nprot.2008.211.	https://davidbioinformatics.nih.gov/summary.jsp RRID:SCR_001881
ClusterProfiler (version 4.10.1)	10.1016/j.xinn.2021.100141	https://bioconductor.org/packages/release/bioc/html/clusterProfiler.html RRID:SCR_016884
fgsea (version 1.28.0)	10.1101/060012	https://bioconductor.org/packages/release/bioc/html/fgsea.html RRID:SCR_020938

Other		

Wheaton dounce tissue grinder	Thermo Fisher Scientific	Cat# 06-435A
CD11b MicroBeads	Miltenyi Biotec	Cat# 130-093-634
LS columns	Miltenyi Biotec	Cat# 130-042-401
KIMBLE dounce tissue grinder	Sigma-Aldrich	Cat# D8938
10x Chromium Controller	10x Genomics	N/A
BZ-X microscope	Keyence	Cat# BZ-X810
SP8 multiphoton microscope	Leica	N/A
CYTEK Cell Sorter	CYTEK	RRID:SCR_023536
Monoject Lavender Stopper Blood Collection Tube	Cardinal Health	Cat# CRD-8881311248

## Highlights

- The microbiome impacts the transcriptional landscape of all major brain cell types
- Discrete gut-resident microbes uniquely modulate brain myeloid cell state
- *E. coli* modulates neural cell states and triggers adaptive neuroimmune activation
- Exposure to *E. coli* exacerbates behavioral and cellular pathologies in 5xFAD mice

## References

[1] Leviatan S, Shoer S, Rothschild D, Gorodetski M, and Segal E. (2022). An expanded reference map of the human gut microbiome reveals hundreds of previously unknown species. Nat. Commun 13, 3863. 10.1038/s41467-022-31502-1. 35790781 PMC9256738

[2] Qin J, Li R, Raes J, Arumugam M, Burgdorf KS, Manichanh C, Nielsen T, Pons N, Levenez F, Yamada T, (2010). A human gut microbial gene catalogue established by metagenomic sequencing. Nature 464, 59–65. 10.1038/nature08821. 20203603 PMC3779803

[3] Scepanovic P, Hodel F, Mondot S, Partula V, Byrd A, Hammer C, Alanio C, Bergstedt J, Patin E, Touvier M, (2019). A comprehensive assessment of demographic, environmental, and host genetic associations with gut microbiome diversity in healthy individuals. Microbiome 7, 130. 10.1186/s40168-019-0747-x. 31519223 PMC6744716

[4] Blackmer-Raynolds L, and Sampson TR (2023). Overview of the Gut Microbiome. Semin. Neurol 43, 518–529. 10.1055/s-0043-1771463. 37562449

[5] Gensollen T, Iyer SS, Kasper DL, and Blumberg RS (2016). How colonization by microbiota in early life shapes the immune system. Science 352, 539–544. 10.1126/science.aad9378. 27126036 PMC5050524

[6] Round JL, and Mazmanian SK (2009). The gut microbiota shapes intestinal immune responses during health and disease. Nat. Rev. Immunol 9, 313–323. 10.1038/nri2515. 19343057 PMC4095778

[7] Thion MS, Low D, Silvin A, Chen J, Grisel P, Schulte-Schrepping J, Blecher R, Ulas T, Squarzoni P, Hoeffel G, (2018). Microbiome Influences Prenatal and Adult Microglia in a Sex-Specific Manner. Cell 172, 500–516.e16. 10.1016/j.cell.2017.11.042. 29275859 PMC5786503

[8] Erny D, Hrabě de Angelis AL, Jaitin D, Wieghofer P, Staszewski O, David E, Keren-Shaul H, Mahlakoiv T, Jakobshagen K, Buch T, (2015). Host microbiota constantly control maturation and function of microglia in the CNS. Nat. Neurosci 18, 965–977. 10.1038/nn.4030. 26030851 PMC5528863

[9] Matcovitch-Natan O, Winter DR, Giladi A, Vargas Aguilar S, Spinrad A, Sarrazin S, Ben-Yehuda H, David E, Zelada González F, Perrin P, (2016). Microglia development follows a stepwise program to regulate brain homeostasis. Science 353, aad8670. 10.1126/science.aad8670. 27338705

[10] Cryan JF, O’Riordan KJ, Cowan CSM, Sandhu KV, Bastiaanssen TFS, Boehme M, Codagnone MG, Cussotto S, Fulling C, Golubeva AV, (2019). The Microbiota-Gut-Brain Axis. Physiol. Rev 99, 1877–2013. 10.1152/physrev.00018.2018. 31460832

[11] Sharon G, Sampson TR, Geschwind DH, and Mazmanian SK (2016). The Central Nervous System and the Gut Microbiome. Cell 167, 915–932. 10.1016/j.cell.2016.10.027. 27814521 PMC5127403

[12] Fang P, Kazmi SA, Jameson KG, and Hsiao EY (2020). The Microbiome as a Modifier of Neurodegenerative Disease Risk. Cell Host Microbe 28, 201–222. 10.1016/j.chom.2020.06.008. 32791113 PMC7430034

[13] Cryan JF, O’Riordan KJ, Sandhu K, Peterson V, and Dinan TG (2020). The gut microbiome in neurological disorders. Lancet Neurol. 19, 179–194. 10.1016/s1474-4422(19)30356-4. 31753762

[14] Boehme M, Guzzetta KE, Bastiaanssen TFS, Van De Wouw M, Moloney GM, Gual-Grau A, Spichak S, Olavarría-Ramírez L, Fitzgerald P, Morillas E, (2021). Microbiota from young mice counteracts selective age-associated behavioral deficits. Nat. Aging 1, 666–676. 10.1038/s43587-021-00093-9. 37117767

[15] Seo DO, O’Donnell D, Jain N, Ulrich JD, Herz J, Li Y, Lemieux M, Cheng J, Hu H, Serrano JR, (2023). ApoE isoform- and microbiota-dependent progression of neurodegeneration in a mouse model of tauopathy. Science 379, eadd1236. 10.1126/science.add1236. PMC9901565 36634180

[16] Harach T, Marungruang N, Duthilleul N, Cheatham V, Mc Coy KD, Frisoni G, Neher JJ, Fåk F, Jucker M, Lasser T, (2017). Reduction of Abeta amyloid pathology in APPPS1 transgenic mice in the absence of gut microbiota. Sci. Rep 7, 41802. 10.1038/srep41802. 28176819 PMC5297247

[17] Minter MR, Hinterleitner R, Meisel M, Zhang C, Leone V, Zhang X, Oyler-Castrillo P, Zhang X, Musch MW, Shen X, (2017). Antibiotic-induced perturbations in microbial diversity during post-natal development alters amyloid pathology in an aged APPSWE/PS1ΔE9 murine model of Alzheimer’s disease. Sci. Rep 7, 10411. 10.1038/s41598-017-11047-w. 28874832 PMC5585265

[18] Minter MR, Zhang C, Leone V, Ringus DL, Zhang X, Oyler-Castrillo P, Musch MW, Liao F, Ward JF, Holtzman DM, (2016). Antibiotic-induced perturbations in gut microbial diversity influences neuro-inflammation and amyloidosis in a murine model of Alzheimer’s disease. Sci. Rep 6, 30028. 10.1038/srep30028. 27443609 PMC4956742

[19] Dodiya HB, Kuntz T, Shaik SM, Baufeld C, Leibowitz J, Zhang X, Gottel N, Zhang X, Butovsky O, Gilbert JA, (2019). Sex-specific effects of microbiome perturbations on cerebral Abeta amyloidosis and microglia phenotypes. J. Exp. Med 216, 1542–1560. 10.1084/jem.20182386. 31097468 PMC6605759

[20] Mezö C, Dokalis N, Mossad O, Staszewski O, Neuber J, Yilmaz B, Schnepf D, de Agüero MG, Ganal-Vonarburg SC, Macpherson AJ, (2020). Different effects of constitutive and induced microbiota modulation on microglia in a mouse model of Alzheimer’s disease. Acta Neuropathol. Commun 8, 119. 10.1186/s40478-020-00988-5. 32727612 PMC7389451

[21] Dodiya HB, Lutz HL, Weigle IQ, Patel P, Michalkiewicz J, Roman-Santiago CJ, Zhang CM, Liang Y, Srinath A, Zhang X, (2022). Gut microbiota-driven brain Abeta amyloidosis in mice requires microglia. J. Exp. Med 219, e20200895. 10.1084/jem.20200895. PMC8647415 34854884

[22] Dodiya HB, Frith M, Sidebottom A, Cao Y, Koval J, Chang E, and Sisodia SS (2020). Synergistic depletion of gut microbial consortia, but not individual antibiotics, reduces amyloidosis in APPPS1–21 Alzheimer’s transgenic mice. Sci. Rep 10, 8183. 10.1038/s41598-020-64797-5. 32424118 PMC7235236

[23] Brain Knowledge Platform. Allen Brain Cell Atlas. https://portal.brain-map.org/atlases-and-data/bkp/abc-atlas.

[24] Van Hove H, Martens L, Scheyltjens I, De Vlaminck K, Pombo Antunes AR, De Prijck S, Vandamme N, De Schepper S, Van Isterdael G, Scott CL, (2019). A single-cell atlas of mouse brain macrophages reveals unique transcriptional identities shaped by ontogeny and tissue environment. Nat. Neurosci 22, 1021–1035. 10.1038/s41593-019-0393-4. 31061494

[25] Yoshida TM, Nguyen M, Zhang L, Lu BY, Zhu B, Murray KN, Mineur YS, Zhang C, Xu D, Lin E, (2025). The subfornical organ is a nucleus for gut-derived T cells that regulate behaviour. Nature 643, 499–508. 10.1038/s41586-025-09050-7. 40437096 PMC12768464

[26] White Z, Cabrera I, Mei L, Clevenger M, Ochoa-Raya A, Kapustka I, Dominguez JR, Zhou J, Koster KP, Anwar S, (2025). Gut inflammation promotes microbiota-specific CD4 T cell-mediated neuroinflammation. Nature 643, 509–518. 10.1038/s41586-025-09120-w. 40533562

[27] Bostick JW, Connerly TJ, Thron T, Needham BD, de Castro Fonseca M, Kaddurah-Daouk R, Knight R, and Mazmanian SK (2025). Genotype and microbiome shape immunity in a sex-specific manner in mouse models of Alzheimer’s disease. Brain Behav. Immun 129, 1014–1027. 10.1016/j.bbi.2025.07.028. 40738263 PMC12490345

[28] Celorrio M, Shumilov K, Ni A, Ayerra L, Self WK, de Francisca NLV, Rodgers R, Schriefer LA, Garcia B, Aymerich MS, (2025). Short-chain fatty acids are a key mediator of gut microbial regulation of T cell trafficking and differentiation after traumatic brain injury. Exp. Neurol 392, 115349. 10.1016/j.expneurol.2025.115349. PMC13135671 40527418

[29] Kline EM, Jernigan JE, Scharer CD, Maurer J, Hicks SL, Herrick MK, Wallings RL, Kelly SD, Chang J, Menees KB, (2025). MHCII reduction is insufficient to protect mice from alpha-synuclein-induced degeneration and the Parkinson’s HLA locus exhibits epigenetic regulation. Sci. Rep 15, 13705. 10.1038/s41598-025-95679-3. 40258905 PMC12012047

[30] Kheirbek MA, and Hen R. (2011). Dorsal vs Ventral Hippocampal Neurogenesis: Implications for Cognition and Mood. Neuropsychopharmacology 36, 373–374. 10.1038/npp.2010.148. 21116266 PMC3055508

[31] Sampson TR, Challis C, Jain N, Moiseyenko A, Ladinsky MS, Shastri GG, Thron T, Needham BD, Horvath I, Debelius JW, (2020). A gut bacterial amyloid promotes alpha-synuclein aggregation and motor impairment in mice. eLife 9, e53111. 10.7554/eLife.53111. PMC7012599 32043464

[32] Chongtham A, Yoo JH, Chin TM, Akingbesote ND, Huda A, Marsh JL, and Khoshnan A. (2022). Gut Bacteria Regulate the Pathogenesis of Huntington’s Disease in Drosophila Model. Front. Neurosci 16, 902205. 10.3389/fnins.2022.902205. PMC9215115 35757549

[33] Liang D, Liu H, Jin R, Feng R, Wang J, Qin C, Zhang R, Chen Y, Zhang J, Teng J, (2023). Escherichia coli triggers alpha-synuclein pathology in the LRRK2 transgenic mouse model of PD. Gut Microbes 15, 2276296. 10.1080/19490976.2023.2276296. PMC10730176 38010914

[34] Chen SG, Stribinskis V, Rane MJ, Demuth DR, Gozal E, Roberts AM, Jagadapillai R, Liu R, Choe K, Shivakumar B, (2016). Exposure to the Functional Bacterial Amyloid Protein Curli Enhances Alpha-Synuclein Aggregation in Aged Fischer 344 Rats and Caenorhabditis elegans. Sci. Rep 6, 34477. 10.1038/srep34477. 27708338 PMC5052651

[35] Wallen ZD, Demirkan A, Twa G, Cohen G, Dean MN, Standaert DG, Sampson TR, and Payami H. (2022). Metagenomics of Parkinson’s disease implicates the gut microbiome in multiple disease mechanisms. Nat. Commun 13, 6958. 10.1038/s41467-022-34667-x. 36376318 PMC9663292

[36] Cattaneo A, Cattane N, Galluzzi S, Provasi S, Lopizzo N, Festari C, Ferrari C, Guerra UP, Paghera B, Muscio C, (2017). Association of brain amyloidosis with pro-inflammatory gut bacterial taxa and peripheral inflammation markers in cognitively impaired elderly. Neurobiol. Aging 49, 60–68. 10.1016/j.neurobiolaging.2016.08.019. 27776263

[37] Liu P, Wu L, Peng G, Han Y, Tang R, Ge J, Zhang L, Jia L, Yue S, Zhou K, (2019). Altered microbiomes distinguish Alzheimer’s disease from amnestic mild cognitive impairment and health in a Chinese cohort. Brain Behav. Immun 80, 633–643. 10.1016/j.bbi.2019.05.008. 31063846

[38] Wang S-S, Li X-H, Liu P, Li J, and Liu L. (2022). The relationship between Alzheimer’s disease and intestinal microflora structure and inflammatory factors. Front. Aging Neurosci 14, 972982. 10.3389/fnagi.2022.972982. PMC9681782 36437994

[39] Khedr EM, Omeran N, Karam-Allah Ramadan H, Ahmed GK, and Abdelwarith AM (2022). Alteration of Gut Microbiota in Alzheimer’s Disease and Their Relation to the Cognitive Impairment. J. Alzheimers Dis 88, 1103–1114. 10.3233/JAD-220176. 35754271

[40] Keren-Shaul H, Spinrad A, Weiner A, Matcovitch-Natan O, Dvir-Szternfeld R, Ulland TK, David E, Baruch K, Lara-Astaiso D, Toth B, (2017). A Unique Microglia Type Associated with Restricting Development of Alzheimer’s Disease. Cell 169, 1276–1290.e17. 10.1016/j.cell.2017.05.018. 28602351

[41] Wang X, Campbell B, Bodogai M, McDevitt RA, Patrikeev A, Gusev F, Ragonnaud E, Kumaraswami K, Shirenova S, Vardy K, (2024). CD8+ T cells exacerbate AD-like symptoms in mouse model of amyloidosis. Brain Behav. Immun 122, 444–455. 10.1016/j.bbi.2024.08.045. 39191349 PMC11409913

[42] Panwar A, Rentsendorj A, Jhun M, Cohen RM, Cordner R, Gull N, Pechnick RN, Duvall G, Mardiros A, Golchian D, (2024). Antigen-specific age-related memory CD8 T cells induce and track Alzheimer’s-like neurodegeneration. Proc. Natl. Acad. Sci. USA 121, e2401420121. 10.1073/pnas.2401420121. PMC11260139 38995966

[43] Kedia S, Ji H, Feng R, Androvic P, Spieth L, Liu L, Franz J, Zdiarstek H, Anderson KP, Kaboglu C, (2024). T cell-mediated microglial activation triggers myelin pathology in a mouse model of amyloidosis. Nat. Neurosci 27, 1468–1474. 10.1038/s41593-024-01682-8. 38937583 PMC11303250

[44] Chen X, Firulyova M, Manis M, Herz J, Smirnov I, Aladyeva E, Wang C, Bao X, Finn MB, Hu H, (2023). Microglia-mediated T cell infiltration drives neurodegeneration in tauopathy. Nature 615, 668–677. 10.1038/s41586-023-05788-0. 36890231 PMC10258627

[45] Laurent C, Dorothée G, Hunot S, Martin E, Monnet Y, Duchamp M, Dong Y, Légeron F-P, Leboucher A, Burnouf S, (2016). Hippocampal T cell infiltration promotes neuroinflammation and cognitive decline in a mouse model of tauopathy. Brain 140, 184–200. 10.1093/brain/aww270. 27818384 PMC5382942

[46] Oakley H, Cole SL, Logan S, Maus E, Shao P, Craft J, Guillozet-Bongaarts A, Ohno M, Disterhoft J, Van Eldik L, (2006). Intraneuronal beta-amyloid aggregates, neurodegeneration, and neuron loss in transgenic mice with five familial Alzheimer’s disease mutations: potential factors in amyloid plaque formation. J. Neurosci 26, 10129–10140. 10.1523/JNEUROSCI.1202-06.2006. 17021169 PMC6674618

[47] Richard BC, Kurdakova A, Baches S, Bayer TA, Weggen S, and Wirths O. (2015). Gene Dosage Dependent Aggravation of the Neurological Phenotype in the 5XFAD Mouse Model of Alzheimer’s Disease. J. Alzheimers Dis 45, 1223–1236. 10.3233/jad-143120. 25697701

[48] Jawhar S, Trawicka A, Jenneckens C, Bayer TA, and Wirths O. (2012). Motor deficits, neuron loss, and reduced anxiety coinciding with axonal degeneration and intraneuronal Aβ aggregation in the 5XFAD mouse model of Alzheimer’s disease. Neurobiol. Aging 33, 196.e29–196.e40. 10.1016/j.neurobiolaging.2010.05.027. 20619937

[49] Serrano-Pozo A, Das S, and Hyman BT (2021). APOE and Alzheimer’s disease: advances in genetics, pathophysiology, and therapeutic approaches. Lancet Neurol. 20, 68–80. 10.1016/s1474-4422(20)30412-9. 33340485 PMC8096522

[50] Morais LH, Schreiber HL, and Mazmanian SK (2021). The gut microbiota–brain axis in behaviour and brain disorders. Nat. Rev. Microbiol 19, 241–255. 10.1038/s41579-020-00460-0. 33093662

[51] Romano S, Wirbel J, Ansorge R, Schudoma C, Ducarmon QR, Narbad A, and Zeller G. (2025). Machine learning-based meta-analysis reveals gut microbiome alterations associated with Parkinson’s disease. Nat. Commun 16, 4227. 10.1038/s41467-025-56829-3. 40335465 PMC12059030

[52] Liang Y, Liu C, Cheng M, Geng L, Li J, Du W, Song M, Chen N, Yeleen TAN, Song L, (2024). The link between gut microbiome and Alzheimer’s disease: From the perspective of new revised criteria for diagnosis and staging of Alzheimer’s disease. Alzheimers Dement. 20, 5771–5788. 10.1002/alz.14057. 38940631 PMC11350031

[53] Vogt NM, Kerby RL, Dill-McFarland KA, Harding SJ, Merluzzi AP, Johnson SC, Carlsson CM, Asthana S, Zetterberg H, Blennow K, (2017). Gut microbiome alterations in Alzheimer’s disease. Sci. Rep 7, 13537. 10.1038/s41598-017-13601-y. 29051531 PMC5648830

[54] Zhuang ZQ, Shen LL, Li WW, Fu X, Zeng F, Gui L, Lü Y, Cai M, Zhu C, Tan YL, (2018). Gut Microbiota is Altered in Patients with Alzheimer’s Disease. J. Alzheimers Dis 63, 1337–1346. 10.3233/JAD-180176. 29758946

[55] Fan K-C, Lin C-C, Chiu Y-L, Koh S-H, Liu Y-C, and Chuang Y-F (2025). Compositional and functional gut microbiota alterations in mild cognitive impairment: links to Alzheimer’s disease pathology. Alzheimers Res. Ther 17, 122. 10.1186/s13195-025-01769-9. 40448221 PMC12123878

[56] Guo M, Peng J, Huang X, Xiao L, Huang F, and Zuo Z. (2021). Gut Microbiome Features of Chinese Patients Newly Diagnosed with Alzheimer’s Disease or Mild Cognitive Impairment. J. Alzheimers Dis 80, 299–310. 10.3233/jad-201040. 33523001

[57] Haran JP, Bhattarai SK, Foley SE, Dutta P, Ward DV, Bucci V, and McCormick BA (2019). Alzheimer’s Disease Microbiome Is Associated with Dysregulation of the Anti-Inflammatory P-Glycoprotein Pathway. mBio 10, e00632–19. 10.1128/mBio.00632-19. PMC6509190 31064831

[58] Hou M, Xu G, Ran M, Luo W, and Wang H. (2021). APOE-epsilon4 Carrier Status and Gut Microbiota Dysbiosis in Patients With Alzheimer Disease. Front. Neurosci 15, 619051. 10.3389/fnins.2021.619051. 33732104 PMC7959830

[59] Liu G-S, Song Y, Yan J-S, Chai Y-J, Zhao Y-F, and Ma H. (2025). Identification of enterotype for patients with Alzheimer’s disease. J. Transl. Med 23, 299. 10.1186/s12967-025-06343-3. 40065353 PMC11892252

[60] Li H, Cui X, Lin Y, Huang F, Tian A, and Zhang R. (2024). Gut microbiota changes in patients with Alzheimer’s disease spectrum based on 16S rRNA sequencing: a systematic review and meta-analysis. Front. Aging Neurosci 16, 1422350. 10.3389/fnagi.2024.1422350. PMC11338931 39175809

[61] Colombo AV, Sadler RK, Llovera G, Singh V, Roth S, Heindl S, Sebastian Monasor L, Verhoeven A, Peters F, Parhizkar S, (2021). Microbiota-derived short chain fatty acids modulate microglia and promote Aβ plaque deposition. eLife 10, e59826. 10.7554/elife.59826. PMC8043748 33845942

[62] Erny D, Dokalis N, Mezö C, Castoldi A, Mossad O, Staszewski O, Frosch M, Villa M, Fuchs V, Mayer A, (2021). Microbiota-derived acetate enables the metabolic fitness of the brain innate immune system during health and disease. Cell Metab. 33, 2260–2276.e7. 10.1016/j.cmet.2021.10.010. 34731656

[63] Ma J, Li M, Bao Y, Huang W, He X, Hong Y, Wei W, Liu Z, Gao X, Yang Y, (2024). Gut microbiota-brain bile acid axis orchestrates aging-related neuroinflammation and behavior impairment in mice. Pharmacol. Res 208, 107361. 10.1016/j.phrs.2024.107361. 39159729

[64] Elkins M, Jain N, and Tükel Ç (2024). The menace within: bacterial amyloids as a trigger for autoimmune and neurodegenerative diseases. Curr. Opin. Microbiol 79, 102473. 10.1016/j.mib.2024.102473. PMC11162901 38608623

[65] Abdel-Haq R, Schlachetzki JCM, Boktor JC, Cantu-Jungles TM, Thron T, Zhang M, Bostick JW, Khazaei T, Chilakala S, Morais LH, (2022). A prebiotic diet modulates microglial states and motor deficits in alpha-synuclein overexpressing mice. eLife 11, e81453. 10.7554/eLife.81453. PMC9668333 36346385

[66] Groh J, Feng R, Yuan X, Liu L, Klein D, Hutahaean G, Butz E, Wang Z, Steinbrecher L, Neher J, (2025). Microglia activation orchestrates CXCL10-mediated CD8+ T cell recruitment to promote aging-related white matter degeneration. Nat. Neurosci 28, 1160–1173. 10.1038/s41593-025-01955-w. 40404995 PMC12148934

[67] Jay TR, Hirsch AM, Broihier ML, Miller CM, Neilson LE, Ransohoff RM, Lamb BT, and Landreth GE (2017). Disease Progression-Dependent Effects of TREM2 Deficiency in a Mouse Model of Alzheimer’s Disease. J. Neurosci 37, 637–647. 10.1523/jneurosci.2110-16.2016. 28100745 PMC5242410

[68] Geva-Zatorsky N, Sefik E, Kua L, Pasman L, Tan TG, Ortiz-Lopez A, Yanortsang TB, Yang L, Jupp R, Mathis D, (2017). Mining the Human Gut Microbiota for Immunomodulatory Organisms. Cell 168, 928–943.e11. 10.1016/j.cell.2017.01.022. 28215708 PMC7774263

[69] Wasén C, Beauchamp LC, Vincentini J, Li S, Leserve DS, Gauthier C, Lopes JR, Moreira TG, Ekwudo MN, Yin Z, (2024). Bacteroidota inhibit microglia clearance of amyloid-beta and promote plaque deposition in Alzheimer’s disease mouse models. Nat. Commun 15, 3872. 10.1038/s41467-024-47683-w. 38719797 PMC11078963

[70] Xia Y, Xiao Y, Wang Z-H, Liu X, Alam AM, Haran JP, McCormick BA, Shu X, Wang X, and Ye K. (2023). Bacteroides Fragilis in the gut microbiomes of Alzheimer’s disease activates microglia and triggers pathogenesis in neuronal C/EBPβ transgenic mice. Nat. Commun 14, 5471. 10.1038/s41467-023-41283-w. 37673907 PMC10482867

[71] Cox LM, Schafer MJ, Sohn J, Vincentini J, Weiner HL, Ginsberg SD, and Blaser MJ (2019). Calorie restriction slows age-related microbiota changes in an Alzheimer’s disease model in female mice. Sci. Rep 9, 17904. 10.1038/s41598-019-54187-x. 31784610 PMC6884494

[72] Casadaban MJ (1976). Transposition and fusion of the lac genes to selected promoters in Escherichia coli using bacteriophage lambda and Mu. J. Mol. Biol 104, 541–555. 10.1016/0022-2836(76)90119-4. 781293

[73] Blackmer-Raynolds L, Lipson LD, Fraccaroli I, Krout IN, Chang J, and Sampson TR (2025). Longitudinal characterization reveals behavioral impairments in aged APP knock in mouse models. Sci. Rep 15, 4631. 10.1038/s41598-025-89051-8. 39920176 PMC11805898

[74] Blackmer-Raynolds L, N Krout IN, and Sampson T. (2024). Object Location Test. protocols.io 10.17504/protocols.io.rm7vzxdo4gx1/v1.

[75] Blackmer-Raynolds L, N Krout IN, and Sampson T. (2024). Y-Maze Protocol. 10.17504/protocols.io.eq2lyjr1mlx9/v1.

[76] Attar A, Liu T, Chan WTC, Hayes J, Nejad M, Lei K, and Bitan G. (2013). A shortened Barnes maze protocol reveals memory deficits at 4-months of age in the triple-transgenic mouse model of Alzheimer’s disease. PLoS One 8, e80355. 10.1371/journal.pone.0080355. PMC3827415 24236177

[77] Blackmer-Raynolds L, N Krout IN, and Sampson T. (2024). Barnes Maze Protocol. protocols.io 10.17504/protocols.io.kxygx3bozg8j/v1.

[78] Hamilton A, N Krout IN, and Sampson T. (2024). Fecal Output Protocol. 10.17504/protocols.io.rm7vzj3j5lx1/v1.

[79] Hamilton A, N Krout IN, and Sampson T. (2024). Fecal Carmine Red Protocol. 10.17504/protocols.io.eq2lywpwwvx9/v1.

[80] Corces MR, Trevino AE, Hamilton EG, Greenside PG, Sinnott-Armstrong NA, Vesuna S, Satpathy AT, Rubin AJ, Montine KS, Wu B, (2017). An improved ATAC-seq protocol reduces background and enables interrogation of frozen tissues. Nat. Methods 14, 959–962. 10.1038/nmeth.4396. 28846090 PMC5623106

[81] Fleming SJ, Chaffin MD, Arduini A, Akkad A-D, Banks E, Marioni JC, Philippakis AA, Ellinor PT, and Babadi M. (2023). Unsupervised removal of systematic background noise from droplet-based single-cell experiments using CellBender. Nat. Methods 20, 1323–1335. 10.1038/s41592-023-01943-7. 37550580

[82] Hao Y, Stuart T, Kowalski MH, Choudhary S, Hoffman P, Hartman A, Srivastava A, Molla G, Madad S, Fernandez-Granda C, (2024). Dictionary learning for integrative, multimodal and scalable single-cell analysis. Nat. Biotechnol 42, 293–304. 10.1038/s41587-023-01767-y. 37231261 PMC10928517

[83] McGinnis CS, Murrow LM, and Gartner ZJ (2019). DoubletFinder: Doublet Detection in Single-Cell RNA Sequencing Data Using Artificial Nearest Neighbors. Cell Syst. 8, 329–337.e4. 10.1016/j.cels.2019.03.003. 30954475 PMC6853612

[84] Zhou Y, Zhou B, Pache L, Chang M, Khodabakhshi AH, Tanaseichuk O, Benner C, and Chanda SK (2019). Metascape provides a biologist-oriented resource for the analysis of systems-level datasets. Nat. Commun 10, 1523. 10.1038/s41467-019-09234-6. 30944313 PMC6447622

[85] Wu T, Hu E, Xu S, Chen M, Guo P, Dai Z, Feng T, Zhou L, Tang W, Zhan L, (2021). clusterProfiler 4.0: A universal enrichment tool for interpreting omics data. Innovation (Camb.) 2, 100141. 10.1016/j.xinn.2021.100141. PMC8454663 34557778

[86] Bray NL, Pimentel H, Melsted P, and Pachter L. (2016). Near-optimal probabilistic RNA-seq quantification. Nat. Biotechnol 34, 525–527. 10.1038/nbt.3519. 27043002

[87] Ritchie ME, Phipson B, Wu D, Hu Y, Law CW, Shi W, and Smyth GK (2015). limma powers differential expression analyses for RNA-sequencing and microarray studies. Nucleic Acids Res. 43, e47. 10.1093/nar/gkv007. 25605792 PMC4402510

[88] Robinson MD, McCarthy DJ, and Smyth GK (2010). edgeR: a Bioconductor package for differential expression analysis of digital gene expression data. Bioinformatics 26, 139–140. 10.1093/bioinformatics/btp616. 19910308 PMC2796818

[89] Zhang Y, Chen K, Sloan SA, Bennett ML, Scholze AR, O’Keeffe S, Phatnani HP, Guarnieri P, Caneda C, Ruderisch N, (2014). An RNA-Sequencing Transcriptome and Splicing Database of Glia, Neurons, and Vascular Cells of the Cerebral Cortex. J. Neurosci 34, 11929–11947. 10.1523/jneurosci.1860-14.2014. 25186741 PMC4152602

[90] Huang DW, Sherman BT, and Lempicki RA (2009). Systematic and integrative analysis of large gene lists using DAVID bioinformatics resources. Nat. Protoc 4, 44–57. 10.1038/nprot.2008.211. 19131956

[91] Sherman BT, Hao M, Qiu J, Jiao X, Baseler MW, Lane HC, Imamichi T, and Chang W. (2022). DAVID: a web server for functional enrichment analysis and functional annotation of gene lists (2021 update). Nucleic Acids Res. 50, W216–W221. 10.1093/nar/gkac194. 35325185 PMC9252805

[92] Korotkevich G, Sukhov V, Budin N, Shpak B, Artyomov MN, and Sergushichev A. (2016). Fast Gene Set Enrichment Analysis. Preprint at bioRxiv. 10.1101/060012.

[93] Blackmer-Raynolds L, and Sampson T. (2024). Protein Extraction for Amyloid Beta Fractionation. 10.17504/protocols.io.j8nlk8ky1l5r/v1.

