# Supplementary material for: Indigenous gut microbes modulate neural cell state and neurodegenerative disease susceptibility
Source: Cell Syst. Author manuscript; Available in PMC 2026 Apr 18. (PMC13091097; doi:10.1016/j.cels.2025.101481)
Supplement: 1 [file NIHMS2126000-supplement-1.pdf]

Cell Systems, Volume *17*

Supplemental information

Indigenous gut microbes modulate neural cell state and neurodegenerative disease susceptibility

Lisa Blackmer-Raynolds, Lyndsey D. Lipson, Anna Kozlov, Aimee Yang, Emily J. Hill, Maureen M. Sampson, Adam M. Hamilton, Isabel Fraccaroli, Sean D. Kelly, Pankaj Chopra, Jianjun Chang, Steven A. Sloan, and Timothy R. Sampson

Supplemental Figure S1. Blackmer-Raynolds et al, 2025

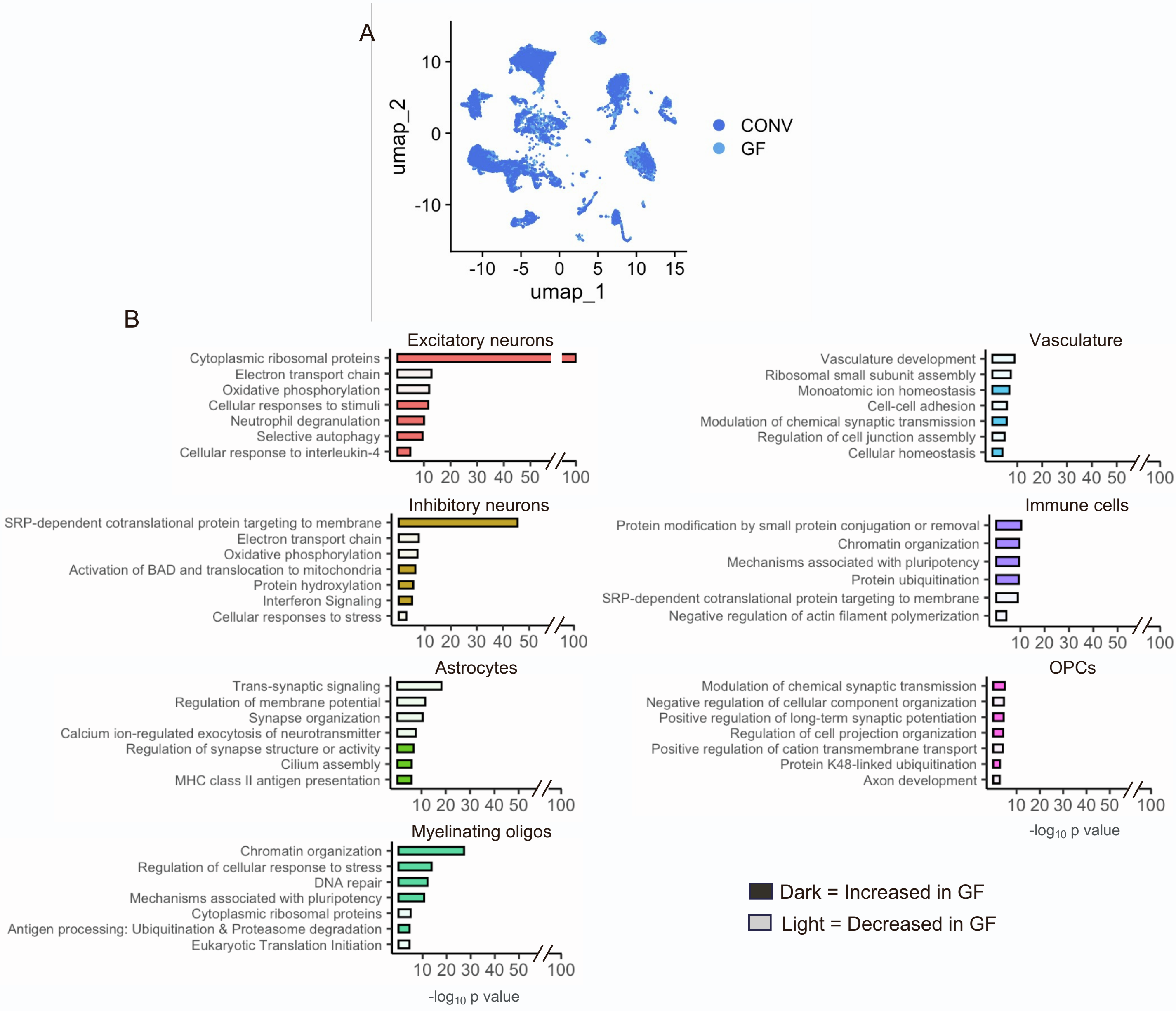

Supplemental Figure S1, *associated with Fig. 1*. Pathways altered in GF mice by cell type. Hippocampal single-nucleus RNA-seq was performed as in Fig. 1 (Supplemental Table S1; NIH GEO accession #GSE289589). **A)** UMAP with nuclei colored by colonization status. **B)** Overrepresentation based pathway analysis was run on every major cell cluster using Metascape (log fold change > |1|, $p < 0.001$). Representative pathways that are increased and decreased in each cell type (and not included in Fig. 1E) are shown. Cells are from 4 mice per treatment group.

Supplemental Figure S2. Blackmer-Raynolds et al, 2025

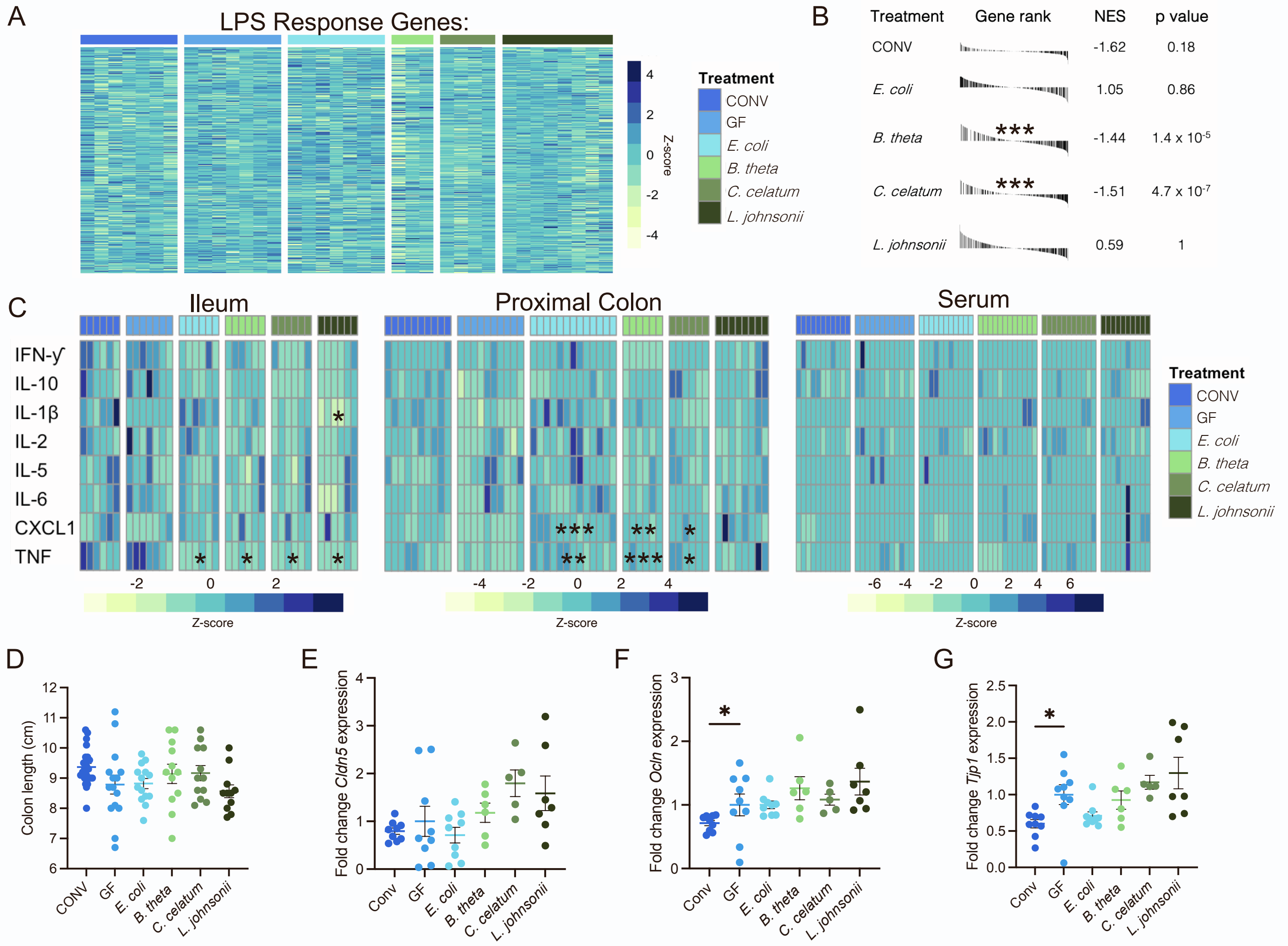

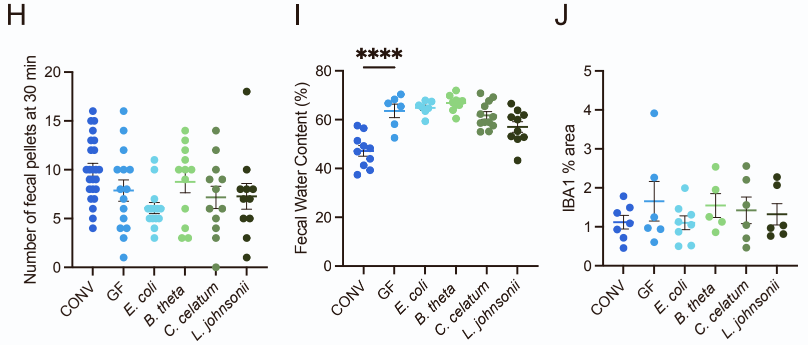

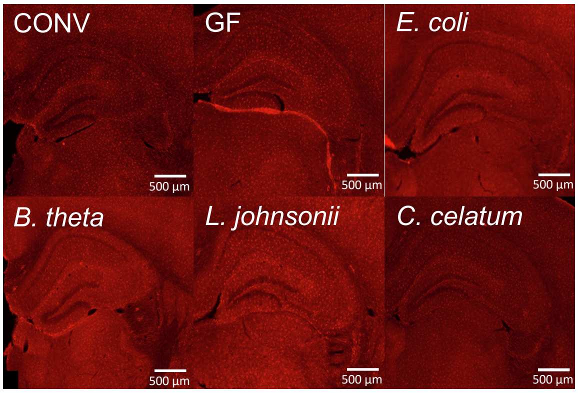

Supplemental Figure S2, *associated with Fig. 2*. Indigenous gut bacteria do not induce robust, widespread inflammation. Bulk RNA-seq of CD11b+ cells performed as in Fig. 2 (Supplemental Table S2; NIH GEO accession #GSE289591). **A)** Heatmap of genes within the "Response to LPS" Gene Ontology pathway (GO:0032496) for each mono-colonization group (represented by z-score). **B)** GSEA for response to LPS GO genes showing normalized enrichment score (NES) and p-value for each group compared to germ-free (GF). **C)** Inflammatory cytokines and chemokines measured in the ileum, proximal colon, and serum (represented by z-score) by multiplex ELISA. **D)** Colon length at the time of sacrifice was recorded as a measure of generalized colonic inflammation. Expression levels relative to GF of tight junction genes **E)** *Cldn5* **F)** *Ocln,* and **G)** *Tjp1* (ZO-1) in the proximal colon measured by qPCR. **H)** Fecal output and **I)** fecal water content was used to assess gastrointestinal function. **J)** IBA1 staining performed in the dorsal hippocampus with IBA1 % area quantification using at least 2 images per mice and 5-7 mice per treatment group. Representative images show one of the images used in analysis per treatment group. n = 3-24. Dots (graphs) or columns (heat maps) represent individual mice. Error bars represent mean ± SEM. Treatment groups were compared to GF using two-way repeated measures ANOVAs (**C**) or one-way ANOVAs **(D-J)** with Dunnett's multiple comparison tests**.** * $p < 0.05$; ** $p < 0.01$; *** $p < 0.001$; **** $p < 0.0001$ compared to GF. Conventionally colonized (CONV).

Supplemental Figure S3.

Blackmer-Raynolds et al, 2025

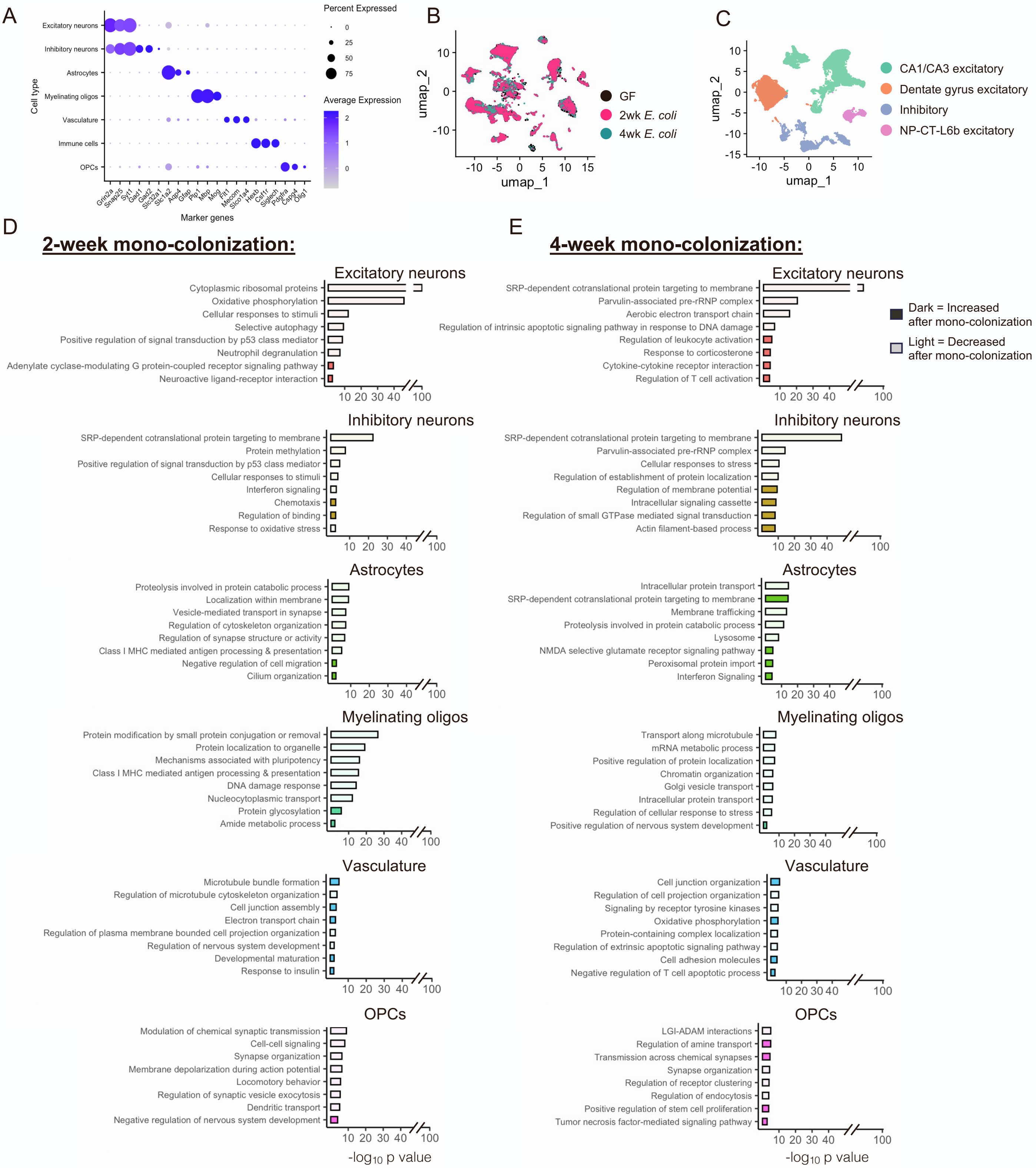

Supplemental Figure S3, *associated with Fig. 3. E.* coli modulates unique biological pathways for each cell type at 2 and 4 weeks. Hippocampal single-nucleus RNA-seq was performed as in Fig. 3 (Supplemental Tables S3 and S4; NIH GEO accession #GSE289589). **A)** Marker genes used for cell type identification. **B)** UMAP showing cell clustering colored by colonization status. **C)** UMAP showing neuronal sub-clustering (results in Supplementary Data Table S4). Overrepresentation based pathway analysis was run on the increased and decreased differentially expressed genes (DEGs; log fold change > |1|, p <0.001) for each cell type after 2 and 4 weeks of mono-colonization. Representative pathways (excluding those shown in Figure 5) for the 2-week timepoint are shown in **D)** and 4-week timepoint are shown in **E).** Data is from 4 mice per treatment.

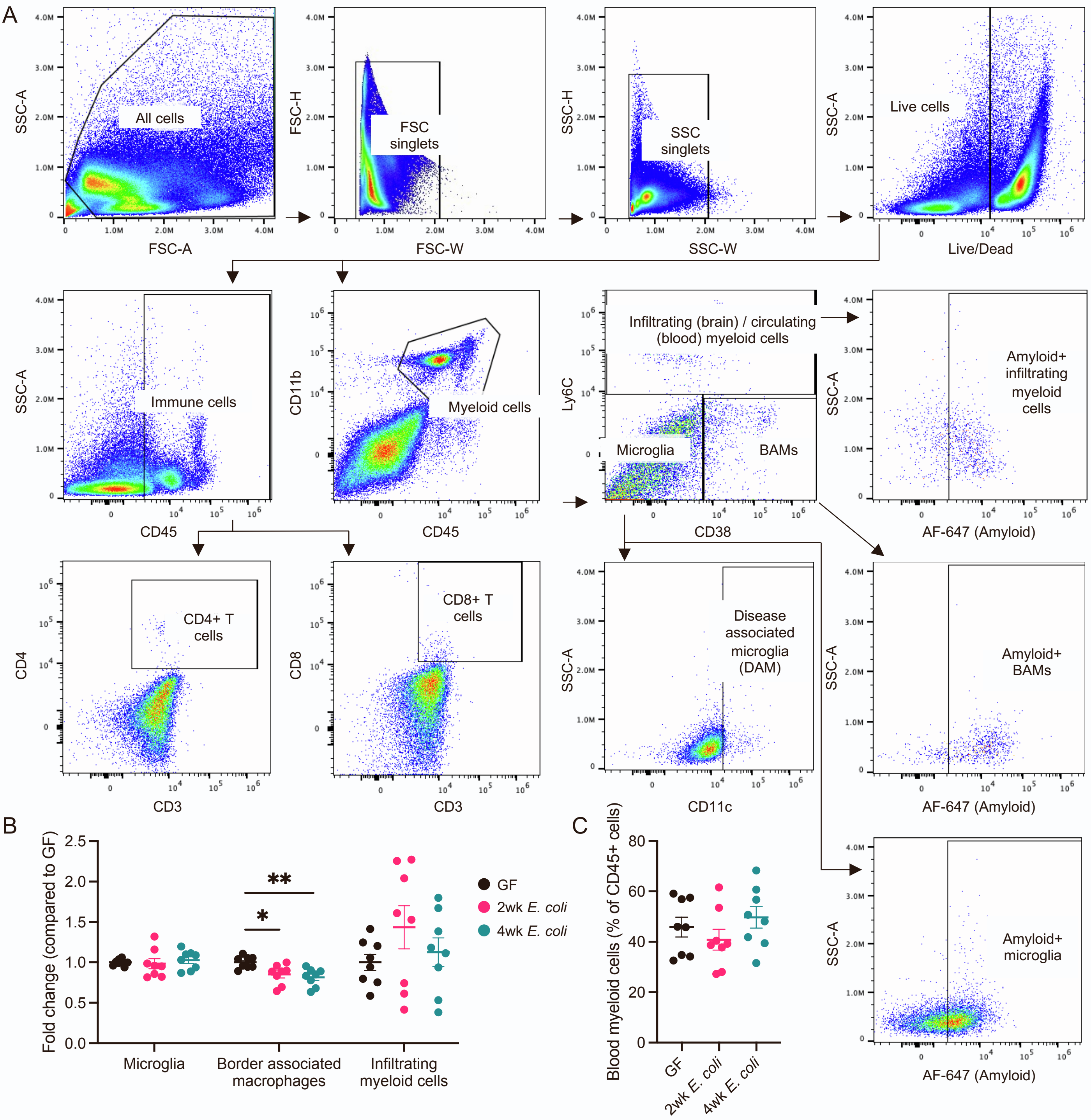

Supplemental Figure S4, *associated with Fig. 4*. Flow cytometry gating and quantification of myeloid cell populations. A) Representative flow gating strategy for brain and blood samples. **B)** Quantification of myeloid cell populations within the brain including microglia (CD45+, CD11b+, Ly6C-, CD38-), border associated macrophages (BAMs; CD45+, CD11b+, Ly6C-, CD38+), and infiltrating (brain) /circulating (blood) myeloid cells (CD45+, CD11b+, Ly6C+). Results are represented as fold change compared to germ-free (GF). **C)** Quantification of myeloid cells (CD45+, CD11b+, Ly6C+) within the blood represented as a percentage of all CD45+ immune cells. In **A)** dots represent individual cells; in **B-C)** dots represent individual mice, error bars represent mean ± SEM. Groups were compared using a two-way repeated measures ANOVA in **B)** or one-way ANOVA in **C)** with Dunnett's test for multiple comparisons. * $p < 0.05$; ** $p < 0.01$.

Supplemental Figure S5. Blackmer-Raynolds et al, 2025

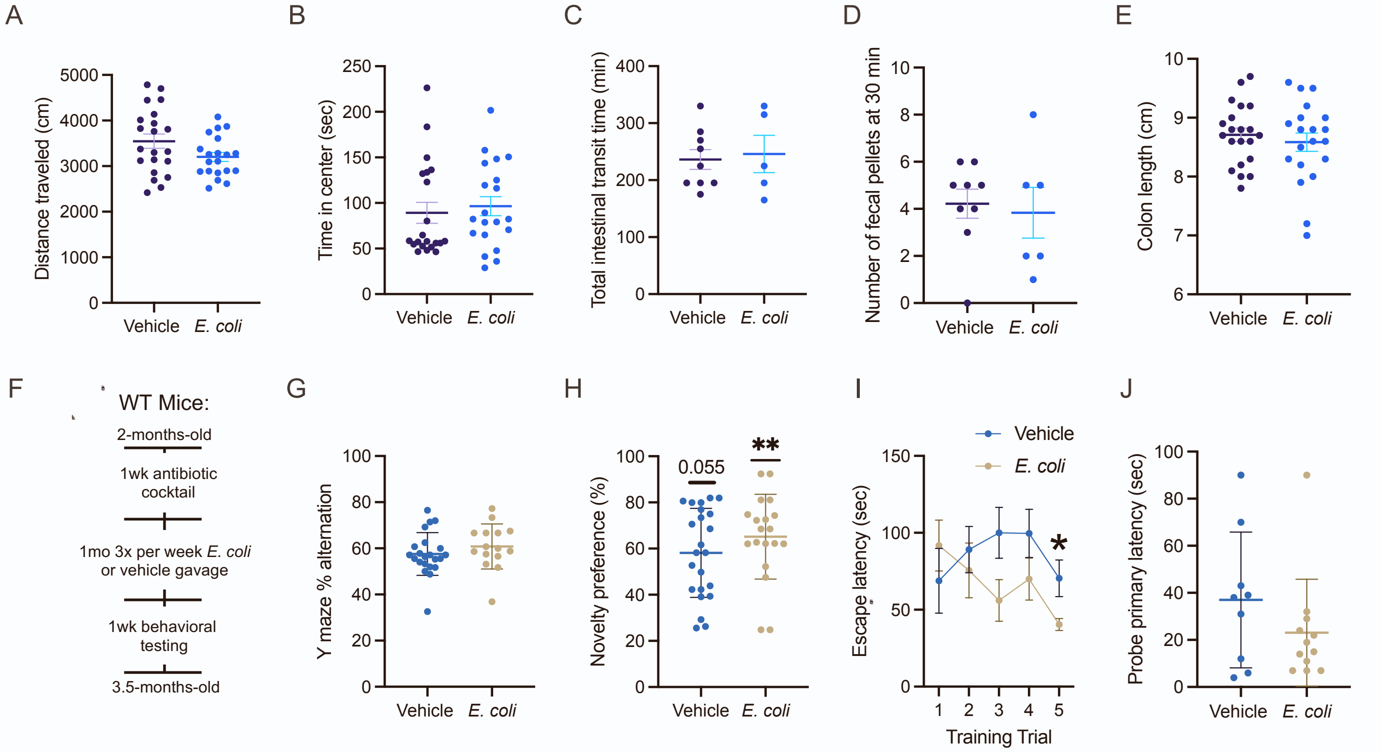

Supplemental Figure S5, *associated with Fig. 5. E. coli* exposure does not induce sickness behaviors in 5xFAD or cognitive impairment in wild-type mice. Vehicle and *E. coli* exposed 5xFAD mice were tested for signs of sickness behavior on a battery of tests. **A)** Motor and **B)** anxiety-like behavior were measured on the open filed test. Gastrointestinal function was measured by **C)** carmine red elution and **D)** total fecal output during a 30-minute period. **E)** Inflammation was measured within the colon via colon length at the time of sacrifice. **F)** Wild-type littermates were tested side by side with 5xFAD mice to see if *E. coli* exposure was sufficient to induce cognitive decline in the absence of familial AD mutations. Performance on the **G)** Y maze, **H)** object location test and **I-J)** Barnes maze. n = 5-23. Dots represent individual mice bars represent mean ± SEM. Groups were compared using T tests except in **H)** where a one sample T test was used to compare to the 50% chance level and **J)** where a two-way repeated measures ANOVA was used. *p < 0.05; ** p < 0.01.

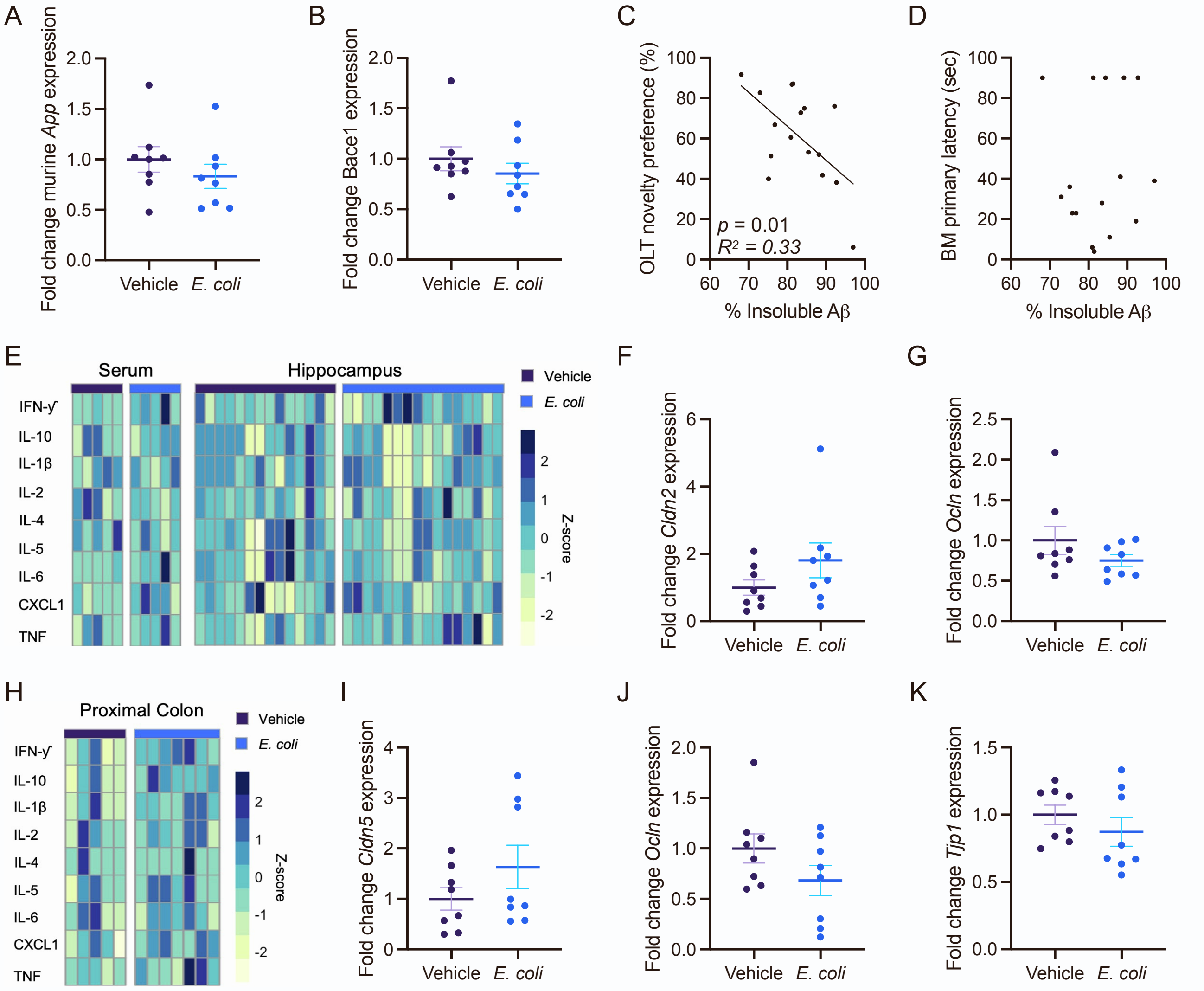

Supplemental Figure S6, *associated with Fig. 5. E. coli* exposure does not induce changes in APP production/processing, cytokines/chemokines, or tight junction protein genes. A) Expression levels of murine *App* and **B)** *Bace1* were measured in the cortex by qPCR. Levels of insoluble amyloid beta were correlated with cognitive performance on the **C)** object location test (OLT) and **D)** Barnes maze (BM) probe trial. **E)** Cytokines and chemokines were measured within the serum and hippocampus by multiplex ELISA. Tight junction genes **F)** *Cldn2* and **G)** *Ocln* measured in the cortex by qPCR. **H)** Cytokines and chemokines (represented by z-score) measured within the proximal colon by multiplex ELISA. Tight junction genes **I)** *Cldn5*, **J)** *Ocln*, and **K)** *Tjp1* (*ZO-1*) measured in the ileum by qPCR. n = 5-21. Dots (or columns) represent individual mice, bars represent mean ± SEM. Treatments were compared using two-tailed t tests except in **C)** and **D)** where correlation was evaluated by Pearson correlation and p value and R^2 were reported for significant correlations and **E)** and **H)** where groups were compared using multiple t tests adjusted for multiple comparisons using two-stage step-up (Benjamini, Krieger, and Yekutieli).
